# Synergy Screening Identifies a Compound That Selectively Enhances the Antibacterial Activity of Nitric Oxide

**DOI:** 10.3389/fbioe.2020.01001

**Published:** 2020-08-25

**Authors:** Wen Kang Chou, Mathini Vaikunthan, Hendrik V. Schröder, A. James Link, Hahn Kim, Mark P. Brynildsen

**Affiliations:** ^1^Department of Chemical and Biological Engineering, Princeton University, Princeton, NJ, United States; ^2^Frick Chemistry Laboratory, Department of Chemistry, Princeton University, Princeton, NJ, United States; ^3^Department of Molecular Biology, Princeton University, Princeton, NJ, United States; ^4^Small Molecule Screening Center, Princeton University, Princeton, NJ, United States

**Keywords:** NO, Hmp, thiazole, high-throughput synergy screening, HTSS

## Abstract

Antibiotic resistance poses a serious threat to global health. To reinforce the anti-infective arsenal, many novel therapeutic strategies to fight bacterial infections are being explored. Among them, anti-virulence therapies, which target pathways important for virulence, have attracted much attention. Nitric oxide (NO) defense systems have been identified as critical for the pathogenesis of various bacteria, making them an appealing therapeutic target. In this study, we performed chemical screens to identify inhibitors of NO detoxification in *Escherichia coli*. We found that 2-mercaptobenzothiazole (2-MBT) can potently inhibit cellular detoxification of NO, achieving a level of inhibition that resembled the effect of genetically removing Hmp, the dominant detoxification enzyme under oxygenated conditions. Further analysis revealed that in the presence of NO, 2-MBT impaired the catalysis of Hmp and synthesis of Hmp and other proteins, whereas in its absence there were minimal perturbations to growth and protein synthesis. In addition, by studying the structure-activity relationship of 2-MBT, we found that both sulfur atoms in 2-MBT were vital for its inhibition of NO detoxification. Interestingly, when 2-mercaptothiazole (2-MT), which lacked the benzene ring, was used, differing biological activities were observed, although they too were NO dependent. Specifically, 2-MT could still prohibit NO detoxification, though it did not interfere with Hmp catalysis; rather, it was a stronger inhibitor of protein synthesis and it reduced the transcript levels of *hmp*, which was not observed with 2-MBT. Overall, these results provide a strong foundation for further exploration of 2-MBT and 2-MT for therapeutic applications.

## Introduction

Antibiotic resistance remains a significant threat to modern medicine (Frieri et al., 2017; The Center for Disease, Dynamics Economics and Policy, 2018). Infections by resistant pathogens contribute to more than 2.8 million hospitalizations and result in more than 35,000 deaths every year in the US alone (CDC, 2019). If left unchecked, antibiotic resistance is projected to cause 10 million annual deaths globally and cost the global economy a total of as much as $100 trillion over the next 30 years (O’Neil, 2014). Furthermore, increasing occurrences of bacteria resistant to last resort antibiotics indicate the precarious state of the antibiotic arsenal (Bratu and Eramo, 2005; Kumarasamy et al., 2010; Meletis, 2016). To address these issues, alternative modalities to treat bacterial infections have been proposed and explored (Clatworthy et al., 2007; Allen et al., 2014; Mahlapuu et al., 2016). These strategies include bacteriophages (Wright et al., 2009; Kutateladze and Adamia, 2010), antimicrobial peptides (Hancock and Sahl, 2006; Mahlapuu et al., 2016), predatory bacteria that prey on pathogens (Kadouri et al., 2013), and agents that target virulence factors that are critical for pathogenesis (Cegelski et al., 2008; Brannon and Hadjifrangiskou, 2016; Dickey et al., 2017; Fleitas Martinez et al., 2019).

Bacterial defenses responsible for neutralizing nitric oxide (NO), which is synthesized by inducible nitric oxide synthase (iNOS) in phagocytes (Missall et al., 2004), have been identified in various pathogens to contribute to virulence (Forrester and Foster, 2012; Robinson et al., 2014). For instance, in *Salmonella enterica*, flavohemoglobin, which is encoded by *hmp* and detoxifies NO by converting it to NO_3_^–^ (Crawford and Goldberg, 1998; Gardner et al., 1998), has been demonstrated to be critical for virulence (Stevanin et al., 2002). In a mouse model, infection with an *hmp* deletion mutant resulted in improved survivability of hosts compared to those infected with wild-type (WT), which indicated that the mutant was less virulent (Bang et al., 2006). This reduction in virulence was confirmed to depend on NO generated in the host, because when the activity of iNOS was blocked by a chemical inhibitor, the *hmp* mutant recovered WT-level virulence (Bang et al., 2006), which validated that the ability to cope with phagocytic NO is closely linked to virulence in *Salmonella*. As another example, the removal of *norV*, which encodes for NO reductase (Hutchings et al., 2002; Spiro, 2012), in enterohemorrhagic *E. coli* (EHEC) reduced bacterial viability inside macrophages (Shimizu et al., 2012), suggesting that the ability to detoxify NO is important for EHEC to withstand immune attack. Moreover, the presence of *norV* was observed to increase the production of Shiga toxin 2 (Shimizu et al., 2012), which has been associated with hemolytic uremic syndrome caused by EHEC (Tozzi et al., 2003), in phagocytized EHEC adding another layer to how nitrosative defenses contribute to bacterial virulence.

Given the importance of NO detoxification to microbial pathogenesis, a potentially feasible strategy to combat bacterial infections centers on disabling their NO defense networks (Stevanin et al., 2002; de Jesus-Berrios et al., 2003; Robinson et al., 2014; Chou and Brynildsen, 2016). However, currently known inhibitors of bacterial NO defenses are not promising drug candidates. Cyanide (CN^–^) has long been recognized as a strong inhibitor of Hmp (Stevanin et al., 2000), but its acute toxicity to humans prevents it from being further developed as a therapeutic agent. Another known inhibitor of Hmp is carbon monoxide (CO) (Gardner et al., 2000), which also presents serious health hazards; although, designing controllable CO-releasing molecules (CORM) is an active area of research (Nobre et al., 2007; Wilson et al., 2015; Flanagan et al., 2018). To address this targeting challenge, Helmick et al. (2005) identified imidazoles as catalytic inhibitors of purified Hmp. Imidazoles can bind to the heme pocket of Hmp, prohibiting the cofactor from being reduced and thereby, blocking downstream reactions (Helmick et al., 2005). However, imidazoles have low permeability across bacterial double membranes and require the co-administration of a membrane-disrupting antibiotic to be effective in Gram-negative bacteria (Pietschmann et al., 2009).

In this study, we performed a chemical screen of 8,320 small molecules to identify selective inhibitors of NO defenses in *E. coli*. From this screen, we identified 2-mercaptobenzothiazole (2-MBT) to be growth inhibitory under aerobic conditions only in the presence of NO. Further analysis revealed that 2-MBT interfered with the catalysis and synthesis of Hmp, the dominant NO-detoxifying enzyme in oxygenated conditions (Gardner et al., 1998; Robinson and Brynildsen, 2013, 2016b). Notably, treating cells with 2-MBT prior to NO exposure reduced their ability to detoxify NO to a level that was comparable to a Δ*hmp* mutant. Investigation of the compound’s structure-activity relationship indicated that both sulfur atoms were crucial for 2-MBT’s inhibitory effects, whereas the benzene ring was important for the inhibition of Hmp catalysis. Specifically, 2-mercaptothiazole (2-MT), which lacks the benzene ring, can block NO detoxification, but does so by reducing *hmp* transcript and Hmp protein levels, rather than inhibition of Hmp catalysis. Overall, this study identified 2-MBT and 2-MT as compounds that selectively enhance the antibacterial activity of NO, elucidated that their mechanisms of action were multi-faceted, and delineated chemical moieties that were important for their activities.

## Materials and Methods

### Strains and Plasmids

All strains and plasmids used in this study are listed in Supplementary Table S1. *E. coli* MG1655 *imp4213*, harboring a truncated *lptD* (Sampson et al., 1989), was generously provided by Thomas Silhavy and used throughout this study. To construct WCMV1, Δ*hmp*:*camR* was introduced into *imp4213* through P1 transduction from a previously generated donor strain (Chou and Brynildsen, 2019). Transductants were selected on Luria-Bertani broth (LB) agar with 10 μg/mL chloramphenicol (CAM), and gene deletion checked by PCR using primers 1 and 3, and 2 and 3 (Supplementary Table S2). Due to the hypersensitivity of *imp4213* to antibiotics (Ruiz et al., 2005), the concentration of CAM used for selection was determined by plating transduction products on LB agar with different concentrations of CAM from 5 to 100 μg/mL and choosing the highest concentration that yielded colonies (10 μg/mL). After confirming *hmp* deletion through colony PCR, retention of the *imp4213* phenotype (absence of suppressor mutants) was verified by plating on LB with 1.5 mg/mL bile salts, which prohibits growth of *imp4213* but allows growth of WT (Ruiz et al., 2005). pWC04 was obtained from a previous study (Chou and Brynildsen, 2019) and pWCMV1 was constructed in this study. These plasmids were separately introduced into *imp4213* Δ*hmp*:*camR* through electroporation, and selected on LB agar with 10 μg/mL kanamycin (KAN). This concentration was also set to the highest KAN concentration that allowed colony formation after transformation. Again, after colony selection from KAN plates, retention of the *imp4213* phenotype was confirmed by plating in the presence of bile salts.

### Media and Chemicals

LB broth was made by dissolving pre-mixed LB (BD Difco, Franklin Lakes, NJ, United States) in Milli-Q water, and LB agar contained the same compositions as that of liquid LB with the addition of 1.5 w/v% agar. MOPS glucose minimal medium (MOPS) was prepared by diluting 10X MOPS buffer (Teknova, Hollister, CA, United States) in Milli-Q water and adding 132 mM K_2_PO_4_ dibasic solution and 20% glucose (Teknova, Hollister, CA, United States) to final concentrations of 132 μM and 10 mM, respectively, followed by sterile-filtering with Millex-MP 0.22 μm filters (Fisher Scientific, Hampton, NH, United States). Antibiotics, KAN and CAM, were purchased from Fisher Scientific (Hampton, NH, United States). DMSO, imidazole, benzimidazole, 2-mercaptobenzothiazole, and its analogs, which included 2-hydroxybenzothiazole, 2-mercaptobenzoxazole, 2-(methylthio)benzothiazole, benzothiazole, thiazole and 2-mercaptothiazole were obtained from Sigma-Aldrich (St. Louis, MO, United States). Cyanide in the form of KCN was purchased from Ricca Chemical (Arlington, TX). DPTA NONOate (DPTA) (Cayman Chemical, Ann Arbor, MI, United States), a NO donor that dissociates to produce 2 molecules of NO per parent compound with a half-life of 3 h at 37°C and is formally known as (Z)-1-[*N*-(3-aminopropyl)-*N*-(3-ammoniopropyl)amino]diazen-1-ium-1,2-diolate, and PAPA NONOate (PAPA) (Cayman Chemical, Ann Arbor, MI, United States), another NO delivering compound that releases 2 molecules of NO per parent compound with a shorter half-life (15 min at 37°C) than that of DPTA and formally referred to as (Z)-1-[*N*-(3-aminopropyl)-*N*-(*n*-propyl)amino]diazen-1-ium-1,2-diolate, were made daily by dissolution in 10 mM NaOH and stored on ice. For calibrating the NO probe, CuCl_2_ was bought from Acros Organics (Waltham, MA, United States), and *N*-(acetyloxy)-3-nitrosothiovaline (SNAP) from Cayman Chemical (Ann Arbor, MI, United States).

### Initial Chemical Screen

**FIGURE 1:**
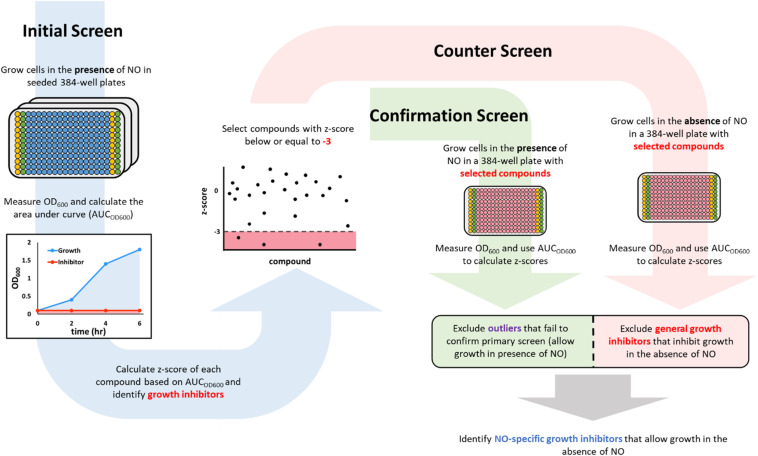
A schematic description of the screening workflow. In the initial screen (blue arrow on the left), 384-well plates were seeded with compounds (blue wells) from a chemical library in the Princeton University Small Molecule Screening Center. The columns on the edges were used to run positive (yellow wells that contained 100 μM KCN) and negative (green wells that contained DMSO). After the addition of cells and DPTA, growth in each well was monitored by measuring OD_600_, which was used to calculate an area under the curve (AUC_OD600_). Compounds that inhibited growth in the presence of NO would have a low AUC_OD600_ (red shaded area), whereas those that allowed growth would yield a high AUC_OD600_ (blue shaded area). Inhibitors from the initial screen (those with a *z*-score < –3) were then passed on to a confirmation screen (green arrow in the middle), in which growth inhibition in the presence of NO was verified, and a counter screen (red arrow on the right), in which growth in the absence of NO was measured. Compounds that failed the confirmation test (green square) or were growth inhibitory in the absence of NO (red square) were discarded. The remaining compounds were identified as NO-dependent growth inhibitors because they allowed growth in the absence of NO, but inhibited growth in the presence of NO at a concentration that could not inhibit growth on its own.

**FIGURE 2:**
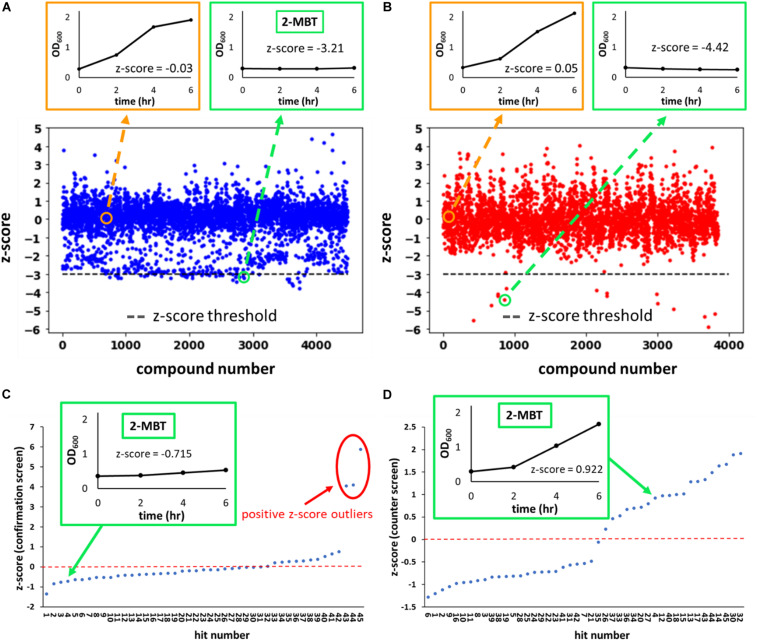
*z*-score from the initial, confirmation and counter screens. In **(A,B)**, the *z*-scores of every compound evaluated during the initial screen are plotted. Compounds were from two sub-libraries: one containing compounds with demonstrated biological activity (biologically active library) **(A)** and the other representing a general small molecule library (generic library) **(B)**. A total of 8,320 compounds were tested in the initial screen, 4,480 of which came from the biologically active library and the remaining 3,840 from the generic library. Representative OD_600_ profiles that either passed (outlined in green) or did not pass (outlined in orange) the *z*-score threshold of –3 (horizontal gray line) were plotted on the top. The growth curve associated with 2-MBT treatment from the initial screen is plotted in the green box in **(A)**. In **(C,D)**, the *z*-scores of hits from the initial screen during the confirmation and counter screens are plotted. In **(C)**, growth inhibition in the presence of NO was verified. Compounds with high *z*-scores allowed growth and were identified as outliers (circle in red). We note that since the confirmation screen only included hits, the *z*-scores between the primary screen and confirmation screen will not be the same. In **(D)**, growth in the absence of NO was measured. NO-specific growth inhibitors would not inhibit growth during this screen, so compounds with the higher *z*-scores allowed relatively better growth. A natural break in the data occurs around a *z*-score of 0 in the counter screen, therefore compounds with *z*-scores > 0 were identified as growth permissive. Of note, out of 20 compounds that had a positive *z*-score in the counter screen, 3 were outliers in the confirmation screen **(C)** and thus were disqualified, and 17 compounds were identified as hits from the screening procedure.

Without NO detoxification, bacterial growth is inhibited by NO (Gardner et al., 1998). Therefore, the initial screen was designed to identify compounds that inhibit growth in the presence of NO (Figure 1). A scrape of cells from a frozen stock of *imp4213* was used to inoculate 1 mL of LB media and grown for 4 h in a shaking incubator maintained at 37°C and 250 rpm. After that, 10 μL of the growing culture was removed and added to 1 mL of fresh MOPS media and grown for 16 h. The overnight culture was then used to inoculate 20 mL of fresh MOPS media in a baffled flask to an OD_600_ of 0.01. Cells were grown to the exponential phase (OD_600_ ∼ 0.25) and harvested for screening. 384-well clear plastic plates (Thermo Fisher Scientific, Waltham, MA, United States) were used for screening. Briefly, the first and last 2 columns (columns 1, 2, 23, and 24) of the plate were used for controls, and each remaining well of the plate contained 0.15 μL of a distinct small molecule dissolved in DMSO, which after addition of the remaining reagents yielded a final concentration of 50 μM. One μL of 3.05 mM KCN, a known inhibitor of the major NO detoxifying enzyme, Hmp, in *E. coli* under oxygenated conditions, was added to every well of the first column (column 1) as the positive control, and 1 μL of DMSO added to the second column as the negative control (column 2). To account for edge effects, we repeated the negative control in the last column (column 24) and the positive control in the second to last column (column 23). After the additions of KCN and DMSO to the outer columns, 28.5 μL of harvested exponential-phase cells were added to each well using a Multidrop Combi^TM^ reagent dispenser (Thermo Scientific, Waltham, MA, United States). Then, 1 μL of 9.91 mM DPTA was manually added to each well using a multi-channel pipette to achieve a final concentration of 325 μM in the control wells and 334 μM in the wells containing small molecules. The slight difference in final DPTA concentrations was due to the different volumes of DMSO and KCN added (1 μL) compared to the small molecules (0.15 μL) added, which was a result of the resolution of the manual multi-channel pipette used in comparison to the small molecule dispenser. Immediately afterward, the OD_600_ in each well was measured using a BioTek Synergy^TM^ H1 Hybrid spectrophotometer. The plate was then sealed with a Breathe-Easy membrane (Sigma-Aldrich, St. Louis, MO, United States) to minimize evaporation and placed in a shaking incubator for growth. At designated time points, the plate was taken out from the incubator and the Breathe-Easy membrane was removed for OD_600_ measurement, after which a new Breathe-Easy membrane was placed on the plate for subsequent growth in the incubator. The OD_600_ profiles were used to calculate the area under curve (AUC_OD600_) during growth, by integrating OD_600_ over time using the trapezoid rule. To assess the quality of data in each plate, AUC_OD600_ values for negative and positive controls were used to calculate z’-factors (Zhang et al., 1999) (Equation 1),

(1) $$z^{\prime }-\mathrm{factor}=1-\frac{3\,\left(\sigma_{p}+\sigma_{n}\right)}{\left|\mu_{p}-\mu_{n}\right|},$$

where μ_p_ and μ_n_ represent the average AUC_OD600_ from the positive control and negative control, respectively, and σ_p_ and σ_n_ the standard deviations of their corresponding means. Plates with z’-factor > 0.5 proceeded to further analysis. For plates that satisfied that criterion, AUC_OD600_ of each compound (AUC_OD600_,_i_) was used to calculate its z-score using Equation 2:

(2) $$z-{\mathrm{score}}_{i}=\frac{{\mathrm{AUC}}_{\mathrm{OD}\,600,i}-{\mathrm{AUC}}_{\mu }}{{\mathrm{AUC}}_{\sigma }},$$

where AUC_μ_ is the average AUC_OD600_ of the plate that the molecule was screened in and AUC_σ_ is the standard deviation of the mean. We identified hits as compounds that had a *z*-score lower than −3 (Figures 2A,B). Of note, the chemical library we used contained 2 subsets–one that contained compounds with demonstrated biological activity (any activity, not specifically against bacteria) and the other that had compounds that had yet to demonstrate biological activity. In total, 4,480 compounds from the biologically active library and 3,840 compounds from the other library were screened.

The concentration of KCN (100 μM in the final volume) was chosen to achieve inhibition of NO detoxification while not completely stopping respiration (Stevanin et al., 2000). The amount of DPTA added was determined by treating cells with a range of DPTA concentrations. For each DPTA concentration examined, growths in the presence and absence of KCN were measured. When DPTA coexisted with KCN, cells could not grow due to inhibition of NO detoxification; whereas, when DPTA was individually treated, cells would resume growth after NO cleared. These growth data were quantified and used to calculate the z’-factor for each concentration of DPTA, using the non-growing condition as the positive control and the growth-permissive condition as the negative control. The threshold for z’-factor was set to 0.5, and among concentrations tested, 325 μM was chosen because it was produced a z’-factor of at least 0.5 in three replicates.

### Counter Screen and Confirmation Screen

Since growth was used as the metric to identify hits from the initial screen, growth inhibitors that are not specific to NO also would have been identified as hits. To eliminate the general growth inhibitors, a counter screen was carried out in the absence of NO. Cells were grown similarly to those grown for the initial screen. The seeded plate used at this step only contained hits from the initial screen. Identical to the initial screen, the first and last 2 columns of the plate were reserved for KCN and DMSO controls, which were set up identically to those used in the initial screen. After exponential-phase cells were added to the wells, instead of treating with DPTA, 1 μL of 10 mM NaOH (DPTA solvent) was added to each well, and growth was quantified with OD_600_ measurements (counter screen). At the same time, an identical set-up was used and DPTA was delivered to provide a second reading on the ability of hits to inhibit growth in the presence of NO (confirmation screen). The resulting growth curves were then used to calculate AUC_OD600_, and the z’-factor was calculated for the confirmation screen (in counter screen both DMSO and KCN grow well). For both plates, z-scores were calculated and plotted (Figures 2C,D). Since samples on these plates were solely comprised of initial hits, z-scores from the confirmation screen would not match those of the initial screen (AUC_μ_ and AUC_σ_ would be different), but rather the z-scores were used to identify positive outliers (e.g., compounds labeled 43, 44, 45), which would have reflected comparably weak growth inhibition in the presence of NO or erroneous identification from the primary screen (Figure 2C). For the counter screen, z-scores were dichotomous with a clear distinction between those with negative z-scores (25 initial hits), which reflected comparably poor growth, and those with positive z-scores (20 initial hits), which reflected comparably better growth (Figure 2D). Three of the initial hits (compounds 43, 44, 45) had positive z-scores in the counter screen, but were not considered further due to their positive outlier z-scores from the confirmation screen. Therefore, from 45 compounds evaluated (initial screen hits), 17 were identified by the confirmation and counter screens as potential NO-specific growth inhibitors.

### Dilution Tests

*imp4213* cells were grown to exponential phase following the procedure described for the chemical screen. A 384-well plate seeded with decreasing concentrations of each of the 17 compounds identified from the counter screen was provided by the Princeton Small Molecule Screening Center. Two wells were assigned to each concentration of the compounds, one was used to measure growth under NO stress and the other growth in the absence of NO. Identical to the initial screen, the first and last columns were used for positive (+KCN) and negative (+DMSO) controls, with positive controls in columns 1 and 23 and negative controls in columns 2 and 24. One μL of 9.91 mM DPTA or the same volume of 10 mM NaOH was then added, and growth monitored by measuring OD_600_. AUC_OD600_s were then calculated by integrating the growth curve. To account for the non-zero AUC_OD600_ baseline, because AUC_OD600_ of a non-growing culture would be larger than 0 due to a constant, non-zero OD_600_, AUC_OD600_ of each compound under each condition was subtracted by the average AUC_OD600_ of the positive controls (+KCN and +DPTA), which was non-growing under our experimental conditions.

### Growth Assay

Cells were grown to exponential phase following procedures described in previous sections. In brief, *imp4213* or WCMV1 cells were grown in 1 mL LB from frozen stocks for 4 h. Then, 10 μL of the growing culture were transferred to 1 mL of fresh MOPS media for 16 h of growth. Following overnight growth, stationary-phase cells were used to inoculate 20 mL of fresh MOPS media in a baffled flask to an OD_600_ of 0.01 and grown to exponential phase (OD_600_ ∼ 0.2). Exponential-phase cells were split and added to several 250 mL baffled flasks, each of which contained 20 mL of fresh MOPS, to an OD_600_ of 0.05, which was consistent with the starting OD_600_ in the bioreactor for NO detoxification assays. Twenty five μL of DMSO or compound of interest dissolved in DMSO were then added to achieve a desired concentration in the flasks. Treated cells were grown in a shaking incubator (37°C, 250 rpm) and growth was monitored hourly by measuring OD_600_, whereas culturability was quantified by extracting 200 μL of the growing culture for CFU/mL measurements every hour.

### Culturability Quantification

To measure culturability during growth, 200 μL of the content from the bioreactor were centrifuged at 15,000 rpm for 3 min and washed by removing 180 μL of the supernatant and resuspending the cell pellet with 180 μL of PBS. Cells in PBS were centrifuged again using the same configuration, followed by the removal of 180 μL of the supernatant. The resulting cell pellet was finally resuspended in 980 μL of PBS and serially diluted in PBS before being plated on an LB-agar plate. Colonies on the plate were counted after 16 h of growth in an incubator maintained at 37°C.

### NO Detoxification Assay

*imp4213* was grown from frozen stock in LB, overnight in MOPS media, and to exponential phase (OD_600_ ∼ 0.2) in MOPS media following the same protocol used for the growth assays. When the target OD_600_ was reached, 8 mL of the exponential-phase culture were removed and added to pre-warmed (37°C) Eppendorf microcentrifuge tubes (Sigma-Aldrich). The tubes were centrifuged at 15,000 rpm for 3 min at room temperature, and supernatant removed. One mL of fresh MOPS media warmed to 37°C was used to resuspend the pellets in all 8 tubes, effectively concentrating the cells. The concentrated culture was then added to a bioreactor consisting of 10 mL fresh MOPS and 25 μL DMSO or compound of interest dissolved in DMSO to achieve an OD_600_ of 0.05. Immediately afterward, DPTA or PAPA dissolved in 10 mM NaOH was added to achieve a concentration of 250 or 34 μM, respectively, in the bioreactor. The bioreactor was a 50 mL Falcon tube (Sigma-Aldrich) that contained a spin bar (1.27 cm) at the bottom spinning at approximately 400 rpm (to ensure well-mixedness), which was placed in a 37°C water bath. Throughout the assay, a NO probe, ISO-NOP 2 mM electrode (World Precision Instruments, Inc.), which uses a proprietary semi-permeable membrane highly selective for NO, was submersed ∼1 cm below surface level in the bioreactor to continuously measure NO concentrations via current readings that were recorded using LabScribe3 software (iWorkx). The probe was calibrated daily using CuCl_2_ and SNAP following procedures described in previous studies (Chou and Brynildsen, 2019). In short, 10 mL of 10 mM CuCl_2_ was added to a 50 mL falcon tube and placed in a water bath kept at 37°C. In the CuCl_2_ solution, a spin bar was added to allow mixing by spinning at 400 rpm and a NO probe was submersed ∼1 cm below the surface to measure NO concentrations. Different concentrations of SNAP were then added sequentially. Under our conditions, we have previously determined that 0.457 equivalents of NO are generated per molecule of SNAP (Chou and Brynildsen, 2019). Using this conversion rate, we created a plot with the concentration of NO produced on the *x*-axis and changes in current (ΔpA) on the *y*-axis, which was used to fit a line. The slope of the fitted equation was then used to convert current readings to NO concentration for experiments conducted on that day (Sivaloganathan et al., 2020). In the cases when CFU/mL data were measured, 200 μL were removed from the bioreactor for plating every 30 min.

### Cell-Free NO Assay

The bioreactor was set up identically to that used for NO detoxification assays. Two hundred and fifty μM of DPTA was added to 10 mL of fresh MOPS media in a bioreactor in the absence of cells. Ten minutes following the DPTA treatment, 25 μL of DMSO or compound of interest were added to the bioreactor. Using a NO probe, NO concentration in the solution was measured continuously throughout the experiment.

### Growth Assay Following NO Exhaustion

The bioreactor was prepared following procedures described previously. Thirty four μM of PAPA or the same volume (34.7 μL) of 10 mM NaOH were added to a bioreactor containing 10 mL of MOPS and 50 μM of 2-MBT or 25 μL of DMSO without cells. Two hours after PAPA or NaOH treatment, which corresponded to the amount of time required for NO to deplete in the cell-free bioreactor when PAPA was used (Supplementary Figure S1), exponential-phase *imp4213* cells were added to the bioreactor to an initial OD_600_ of 0.05. OD_600_ was then measured hourly to monitor bacterial growth.

### LC-MS Following NO Exhaustion in Cell-Free Bioreactor

In a cell-free bioreactor consisting of 10 mL of MOPS and 50 μM of 2-MBT or equivalent volume (25 μL) of DMSO, 34 μM of PAPA or 34.7 μL of 10 mM NaOH were added. After 2 h of treatment, samples were removed for analysis via liquid chromatography-mass spectrometry (LC-MS). For LC-MS measurements, reaction solutions (10 μL) were injected onto a Zorbax 300SB-C18 column (2.1 mm × 50 mm, 3.5 μm) using a 1260 Infinity II system (Agilent). The mobile phase consisted of a mixture of water and acetonitrile (HPLC grade) both containing 0.1% (v/v) formic acid. Separation was achieved during 25 min runs by applying a flow rate of 0.5 mL/min and a linear, binary solvent gradient as follows. From 0 to 1 min, the solvent was maintained as 90% water/10% ACN. This was followed by linear ramping to 50% water/50% ACN from 1 min- 20 min and subsequent ramping to 10% water/90% ACN (20–25 min). The separated species were analyzed with a connected Agilent 6530 Accurate Mass Q-TOF mass spectrometer using electrospray ionization in positive mode. Extracted ion chromatograms (EIC) were generated by selecting a window around *m/z* 167.9, the monoisotopic mass of [2-MBT + H]^+^ is 167.992 Da.

### Inhibition Assay After NO Clearance

Cells were grown to exponential phase, harvested via centrifugation, and treated with DPTA in a bioreactor following the procedures described in the preceding sections, with the exception that the bioreactor did not contain any compound of interest or DMSO before DPTA treatment. One hour after the addition of DPTA, which was after NO had been cleared by cells, one of the following compounds was added to the bioreactor: 100 μM KCN, 100 μg/mL CAM, 50 μM 2-MBT, 50 μM 2-MT, or equivalent volumes of their respective solvents (25 μL DMSO or 10 μL ethanol). The time of NO clearance was determined as when the NO concentration in the bioreactor fell below 0.2 μM, which corresponded to ∼57 min after DPTA treatment.

### Promoter Activity Assay

*imp4213*Δ*hmp* harboring pUA66, pWCMV1, or pWC04 was grown to exponential phase following procedures used for NO detoxification assays, with the exception that 10 μg/mL of KAN was included throughout growth for plasmid retention. In pUA66, *gfp_*mut*2_* is devoid of a promoter; whereas, *gfp*_*SF*_ is expressed from P*_*T*5_* with the addition of IPTG in pWCMV1, and *gfp*_*SF*_ from the native promoter of *hmp* (P*_*hmp*_*) in pWC04. After centrifugation and resuspension, exponential-phase cells were added to a bioreactor containing 10 mL MOPS, 10 μg KAN and one of the following compounds: 50 μM 2-MBT, 50 μM 2-MT, or 25 μL DMSO (equivalent volume of solvent). Immediately afterward, 250 μL of the culture were fixed and another 250 μL were removed and mixed with 500 μL RNAprotect Bacteria Reagent (Qiagen) for 5 min at room temperature (time 0 measurements). Then 250 μM of DPTA or equal volume of NaOH were added to the bioreactor. Of note, when the *T*_5_ promoter was used, 2 mM IPTG was added quickly after DPTA/NaOH treatment to induce *gfp*_*SF*_ expression. At designated times, 250 μL of cells in the bioreactor were removed for fixation and another 250 μL withdrawn for RNAprotect treatment. After 5 min in RNAprotect, samples were centrifuged at 5,000 *g* for 10 min. Then, the supernatant was discarded, and cell pellet stored at −80°C until qPCR. In the case when CFU/mL were measured, 200 μL were removed from the bioreactor for plating at designated times.

### Cell Fixation and Flow Cytometry

Cells removed for fixation were immediately centrifuged for 2 min at 15,000 rpm. Supernatant was discarded and the remaining cell pellet was resuspended in 250 μL of 4% paraformaldehyde (PFA) dissolved in PBS. The solution was incubated at room temperature for 30 min, after which fixed cells were washed once by spinning at 15,000 rpm for 2 min and resuspending in 250 μL PBS. Following the wash, cells were centrifuged again, resuspended in PBS and stored at 4°C until fluorescence quantification. GFP quantification was performed using an LSR II flow cytometer (BD Biosciences) by measuring the FITC-A of 100,000 cellular events, which were recorded using the FACSDiva software provided by the manufacturer. Gating for cell size was based on fixed WT cells, and fluorescence detection was calibrated based on fixed WT cells (non-fluorescent control) and fixed WCMV1 transformed with pWCMV1 with *gfp*_*SF*_ expression fully induced overnight for 16 h (highly fluorescent control). All measurements directly compared in our analyses were obtained on the same day from the same fluorescence calibration.

### qPCR

mRNA was extracted from samples stored at −80°C using RNeasy Mini kit (Qiagen, Germantown, MD, United States) following manufacturer’s protocol. To account for any loss of mRNA during extraction, 50 ng of *phzM* mRNA were added to every sample at the beginning of extraction. Briefly, *phzM* mRNA was prepared by linearizing the *phzM* harboring pET11a plasmid using the restriction enzyme *Eco*RI (New England Biolabs, Ipswich, MA, United States) and performing *in vitro* transcription on the linearized plasmid using TranscriptAid T7 high yield transcription kit (Thermo Fisher Scientific, Waltham, MA, United States). Synthesized mRNA was then purified using RNeasy Mini kit (Qiagen, Germantown, MD, United States). Recovered mRNA from cellular samples was converted to cDNA using Taqman reverse transcription kit (Thermo Fisher Scientific, Waltham, MA, United States) using random hexamers. To quantify mRNA abundances of *gpf*_*SF*_ and *phzM*, qPCR was performed on a 0.1 mL MicroAMP fast optical 96-well reaction plate (Thermo Fisher Scientific, Waltham, MA, United States) using appropriate primers (Supplementary Table S2) and SYBR green (Thermo Fisher Scientific, Waltham, MA, United States) as the reporter for DNA synthesis. The loaded plate was seeded into ViiA 7 real-time PCR system (Thermo Fisher Scientific, Waltham, MA, United States) for 40 cycles. The cycle threshold (Ct) values of the controls (linearized pWCMV1 for *hmp* and linearized pET11a-*phzM* for *phzM*) were used to fit a linear relationship with the concentrations of the plasmids added. This linear equation was then used to calculate the mRNA concentration in each well. In each sample, the abundance of *gfp*_*SF*_ mRNA was normalized by the measured level of *phzM* mRNA.

## Results

### Screening Strategy

Bacterial growth is prohibited by NO at or above micromolar concentrations (Thomas et al., 2008; Schairer et al., 2012). Only cultures with active detoxification systems can convert NO to non-toxic forms (e.g., NO_3_^–^ in *E. coli* under aerobic conditions) (Gardner and Gardner, 2002; Chou and Brynildsen, 2016; Robinson and Brynildsen, 2016a) and reduce its concentration to levels that allow growth. Therefore, to begin to identify inhibitors of NO detoxification, two screening steps were used. The first identified compounds that could inhibit bacterial growth in the presence of NO, whereas the second tested hits from the first screen for their capacity to inhibit bacterial growth in the absence of NO. Overall, we sought to identify compounds that impaired growth in the primary screen (NO present) that could not inhibit growth in the counter screen (NO absent).

### Initial Screen in the Presence of NO

To facilitate active compound identification, we used the strain *imp4213* throughout this study. This strain has a truncated *lptD* on the chromosome that results in a compromised outer membrane (Sampson et al., 1989), thereby allowing extracellular compounds to permeate more easily (Ruiz et al., 2005). Use of such mutants in chemical screening has been done previously (Howe et al., 2015).

The screen was performed in 384-well plates where the boundary columns of each plate were reserved for positive (100 μM KCN) and negative (DMSO) controls; each remaining well contained 50 μM of a compound from the library. Cells were grown to exponential phase (OD_600_ ∼ 0.25) and added to the plates. Immediately afterward, DPTA, the NO donor, was added to each well. Growth in the presence of NO was monitored by OD_600_ measurements at time points over 6 h. Area under the OD_600_ curve (AUC_OD600_) from each well was then calculated to quantify the extent of growth (Figure 1). Growth data from KCN and DMSO controls were used to calculate the z’-factor (Equation 1), which quantified the quality of data of each plate by assessing the statistical distance between the controls. Plates that had a z’-factor below the threshold of 0.5 were discarded. For each of the remaining plates, z-scores of each compound on the plate were calculated to assess its relative ability to influence growth against the average of the plate. Hits were identified as compounds that had a z-score of −3 or lower (Figure 1). A z-score below −3 indicated that in the presence of the compound, cellular growth, quantified using AUC_OD600_, was at least 3 standard deviations below the average growth on the plate (Figures 2A,B). Out of 8,320 compounds screened, 45 were identified as hits. As expected, the majority of them were from the subset of the library with demonstrated biological activity (Supplementary Figure S2A). Further analysis revealed that the average AUC_OD600_ from the biologically active library was lower than that from the generic library (Supplementary Figure S2B), suggesting that compounds with demonstrated activity were more likely to negatively affect growth in this assay.

### Counter Screen in the Absence of NO, Confirmation Screen, and Dilution Tests

By using growth as the metric for defining hits in the initial screen, both NO detoxification inhibitors and general growth inhibitors would be identified. To distinguish general growth inhibitors from compounds that inhibited growth only in the presence of NO, exponential-phase cells were grown with hits in the absence of NO in a 384-well plate (counter screen). Under this condition, molecules that specifically impaired NO detoxification would allow growth, whereas those that targeted essential cellular processes would still inhibit growth (Figure 1). In addition, an identical 384-well plate was grown in the presence of NO as a confirmation step for the initial screen hits (confirmation screen). z-scores from these plates were inspected and potential NO detoxification inhibitors were identified as those compounds with positive z-scores in the counter screen, which did not yield positive z-score outliers in the confirmation screen (Figures 2C,D). With these criteria, 17 of the hits from the initial screen were identified as potential compounds with selective inhibition of *E. coli* NO defenses.

**FIGURE 3:**
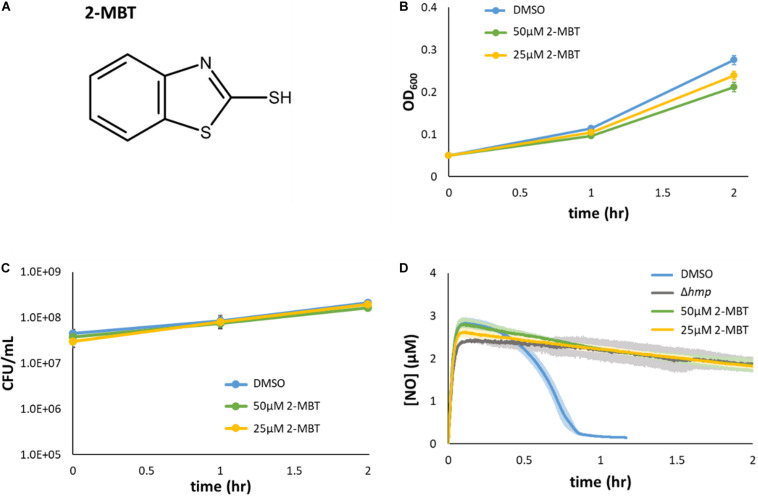
Validation of 2-MBT activity. **(A)** The chemical structure of 2-mercaptobenzothiazole (abbreviated as 2-MBT) was drawn using ACD/ChemSketch (ACD/ChemSketch, 2020). **(B,C)** Bacteria were grown in liquid media in the absence of NO with different concentrations of 2-MBT. Growth **(B)** and culturability **(C)** were monitored hourly. Solid points represent the mean of at least 3 independent replicates and the error bars reflect the standard error of the means. **(D)** [NO] following compound and DPTA addition at time = 0 was continuously measured using a NO probe. Quickly after the addition of DPTA, [NO] peaked at ∼3 μM. The time of NO clearance was identified as when [NO] ≤ 0.2 μM, which corresponded to ∼0.95 h for cells treated with DMSO. In Δ*hmp*, the dominant NO detoxification enzyme under oxygenated conditions is absent, and therefore, the gray line corresponds to negligible cellular detoxification of NO. Solid lines are the average measurements of at least 3 biological replicates, and the shaded regions around the lines are the standard errors of the means.

To deduce strengths of inhibition, we serially diluted potential inhibitors in a 384-well plate, with each concentration occupying 2 wells. One well was used to grow cells under NO stress, and the other in the absence of NO. Growth in terms of AUC_OD600_ was compared between ±NO conditions, with the aim of identifying compounds with large differences across a wide range of concentrations. Among the compounds examined, 2-mercaptobenzothiazole (2-MBT) (Figure 3A), which was from the biologically active library, consistently exhibited strong inhibition of growth under NO stress while permitting growth under stress-free conditions (Supplementary Figure S3). As a result, 2-MBT was chosen for further analysis.

### Validation of 2-MBT as an NO-Dependent Inhibitor

To validate that 2-MBT was not a growth inhibitor under normal growth conditions (confirm counter screen results), growth assays were performed in flasks with varying concentrations of 2-MBT. The results verified that the concentration of 2-MBT used in the initial screening (50 μM) allowed for growth in NO-free environments, achieving an OD_600_ that was 77% of the DMSO-only treated control after 2 h (Figure 3B). When the 2-MBT concentration was dropped to 25 μM, growth by 2 h was 87% of the control. In addition, we measured CFU/mL and found that 2-MBT did not impact culturability in the absence of NO (Figure 3C). These data corroborate the screening results that suggested 2-MBT only had a minor effect on normal growth.

The inhibitory effects of 2-MBT on NO detoxification were subsequently evaluated. Exponential-phase bacteria were exposed to NO in a bioreactor containing fresh MOPS media and either 2-MBT or DMSO. After DPTA treatment at *t* = 0, the [NO] in the system rapidly peaked within 7 min and was cleared from the system quickly (approximately 46 min) in the solvent-only control (DMSO) (Figure 3D). The time of NO clearance was assessed as the time at which [NO] dropped below 0.2 μM in the bioreactor (Robinson and Brynildsen, 2016b). When 2-MBT was used for treatment, the [NO] dynamics were close to those of Δ*hmp* (Figure 3D), which suggested that cellular detoxification was greatly compromised. Notably, even when [2-MBT] was reduced by half, the strength of inhibition was unaffected, which demonstrated the potency of 2-MBT as an inhibitor of NO detoxification in *E. coli*. Additionally, to ensure that the effects observed were independent of the NO donor, we performed NO detoxification assays using a structurally different NO donor, PAPA. When treated with PAPA, 2-MBT still inhibited NO detoxification to an extent similar to Δ*hmp*, whereas DMSO-treated cells cleared NO rapidly (Supplementary Figure S4). These data suggested that the effects of 2-MBT on bacterial NO detoxification were independent of the NO donor.

### Reactivity of 2-MBT With NO in Cell-Free Conditions

**FIGURE 4:**
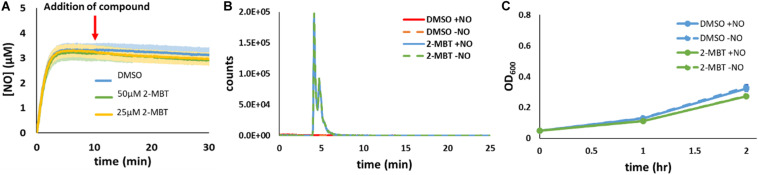
Reactivity of 2-MBT to NO in the absence of cells. **(A)** [NO] in the bioreactor in the absence of cells was measured. At *t* = 0, DPTA was added and after 10 min different concentrations of 2-MBT or the same volume of DMSO was added to the bioreactor. The solid lines represent the averages from at least 3 biological replicates, and the shaded regions represent the standard errors of the means. **(B)** 34 μM of PAPA or 34.7 μL of 10 mM NaOH were added to a bioreactor containing MOPS and 50 μM of 2-MBT or DMSO in the absence of bacteria. After 2 h, which correspond to the amount of time it took for NO to deplete under cell-free settings (Supplementary Figure S1), samples from the bioreactor were withdrawn for LC-MS analysis. Representative extracted-ion chromatograms (EIC) at m/z of 167.9 of 3 replicate experiments are plotted. Representative total-ion current chromatogram (TIC) is provided in Supplementary Figure S5 and no significant signal intensities for the 2-MBT dimer or other oxidized species were observed in the mass spectra. **(C)** Bacterial growth in the bioreactor containing MOPS and 2-MBT or DMSO that had or had not been exposed to NO was measured hourly. The solid dots represent the average OD_600_ from 3 biological replicates, and the error bars the error of the means.

To assess whether 2-MBT was influencing NO detoxification by reacting with NO (e.g., to form a toxic by-product), experiments were performed with 2-MBT and DPTA in the absence of cells. After [NO] in the bioreactor became relatively stable, which corresponded to 10 min after DPTA treatment, 2-MBT or the same volume (25 μL) of DMSO were added to the bioreactor. Results indicated that there was no significant difference in [NO] between samples that contained 2-MBT and those treated with just DMSO (Figure 4A). In addition, LC-MS analyses revealed that there was no depletion of 2-MBT when NO was present and no evidence of the formation of new compounds (Figure 4B and Supplementary Figure S5). Furthermore, when exponential-phase *imp4213* were added to a bioreactor containing MOPS and 2-MBT that had or had not contained NO, cells grew readily and there was no significant difference in growth between the different conditions (Figure 4C). Collectively, these results suggest that 2-MBT and NO do not react appreciably nor do they form toxic products in cell-free media. Rather, the impact of 2-MBT on [NO] dynamics requires the concurrent presence of cells.

### Structure Activity Relationship

**FIGURE 5:**
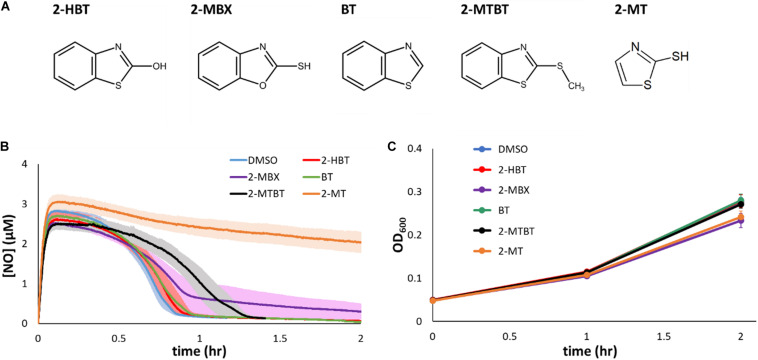
Chemical analogs of 2-MBT and their effects on NO detoxification and growth. **(A)** 2-hydroxybenzothiazole is abbreviated as 2-HBT, 2-mercaptobenzoxazole as 2-MBX, benzothiazole as BT, 2-(methylthio)benzothiazole as 2-MTBT, and 2-mercaptothiazole as 2-MT. Structures were drawn using ACD/ChemSketch (ACD/ChemSketch, 2020). **(B)** [NO] in the bioreactor was monitored following treatments of DPTA and analogs of 2-MBT or DMSO. Solid lines represent the average measurements from at least 3 biological replicates and the shading the standard errors of the means. **(C)** Bacterial growth in the presence of 50 μM of different 2-MBT analogs under NO-free environment was monitored by measuring OD_600_ at designated times. Solid points are the means of at least 3 independent experiments, and the error bars are the standard errors of those means.

Given the ability of 2-MBT to inhibit NO detoxification, we sought to explore the structure activity relationship between 2-MBT and its impact on *E. coli* NO consumption. To accomplish that, analogs of 2-MBT (Figure 5A) were assessed for their abilities to impair NO detoxification. These analogs included 2-hydroxybenzothiazole (2-HBT), which had a hydroxyl group rather than a mercapto group attached to the thiazole ring; benzothiazole (BT), which lacked the mercapto group; 2-(methylthio)benzothiazole (2-MTBT), which had a methyl group in place of the hydrogen of the mercapto group (thereby preventing disulfide bond formation); 2-mercaptobenzoxazole (2-MBX), which had an oxazole ring instead of a thiazole ring; and 2-mercaptothiazole (2-MT), which lacked the benzene ring. Immediately after the addition of each analog to the bioreactor, DPTA was added and NO detoxification assays were carried out.

Treatment of 2-HBT did not delay NO detoxification (Figure 5B), suggesting that the mercapto group is essential to the ability of 2-MBT to inhibit NO detoxification. Additional experiments with the treatment of BT confirmed the importance of the mercapto group to inhibition (Figure 5B). Moreover, when the sulfur atom of the mercapto group was present, but its ability to form disulfide bonds was removed by substituting the hydrogen for a methyl group (2-MTBT), inhibition of NO detoxification was largely lost (Figure 5B).

To evaluate whether the sulfur atom within the thiazole ring of 2-MBT was required for inhibition of NO detoxification, 2-MBX was added to cell cultures prior to the introduction of NO. Treatment with 2-MBX minimally perturbed NO detoxification by *E. coli* (Figure 5B), which suggested that the sulfur atom in the thiazole ring was important for inhibition of NO defenses. We further assessed the necessity of the benzene ring of 2-MBT with its analog 2-MT. When cells were treated with 2-MT, their NO-detoxifying capabilities became greatly reduced (Figure 5B), and reached a level of inhibition similar to that observed with 2-MBT treatment. These data suggested that the benzene ring was not required for inhibition of NO detoxification. Moreover, to validate that any effect on NO detoxification by these analogs was NO-specific, growth assays were carried out by treating exponential-phase cells with each of the analogs and monitoring growth in the absence of NO (Figure 5C). Growth data showed that analogs did not inhibit growth appreciably on their own. Overall, these results show that both sulfur atoms and the mercapto group of 2-MBT are important for the inhibition of NO detoxification, whereas the benzene group is dispensable.

### Survivability of *imp4213* Under NO Stress

**FIGURE 6:**
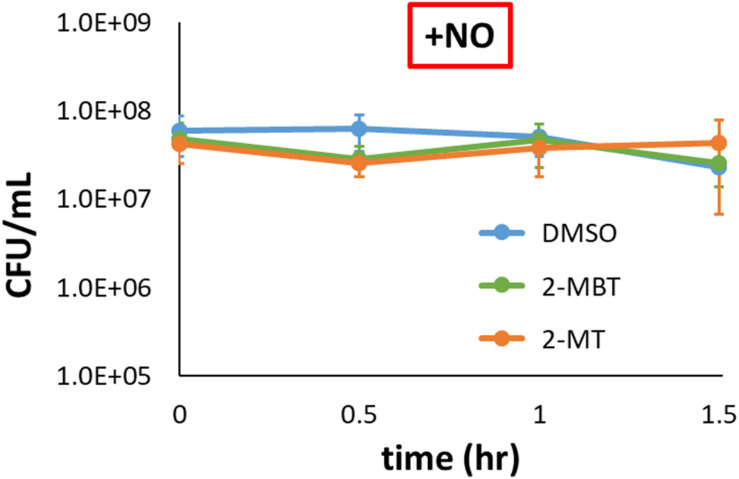
Culturability of *imp4213* in the presence of 2-MBT or 2-MT during NO stress. Bacterial survival under NO in the presence of 50 μM of 2-MBT, 50 μM of 2-MT, or 25 μL of DMSO was quantified by measuring CFU/mL every 30 min. NO was provided by DPTA in these assays. The solid circles are the mean CFU/mL measurements from 3 independent replicates, and the error bars represent the standard errors of the means.

We sought to investigate how 2-MBT and 2-MT mechanistically inhibit NO detoxification. NO is generally bacteriostatic but can be bactericidal in certain genetic backgrounds (e.g., Δ*dksA*) (Chou and Brynildsen, 2019). Since NO detoxification requires reducing equivalents, cell death might underlie the inhibition of NO detoxification we observed in the presence of 2-MBT and 2-MT. To assess this possibility, culturability of *imp4213* was measured in the presence of NO when treated with DMSO, 50 μM of 2-MBT, or 50 μM of 2-MT. CFU/mL data were constant over the observation period for all samples (Figure 6), which suggested that 2-MBT and 2-MT do not impair NO detoxification by stimulating bacterial killing.

### Inhibition Assay After NO Clearance

**FIGURE 7:**
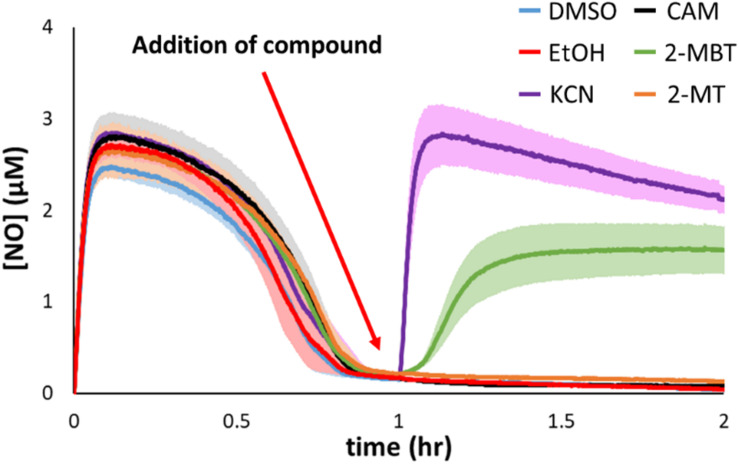
Inhibitory effects of 2-MBT and 2-MT on Hmp catalysis. Following DPTA treatment at time = 0, [NO] was continuously monitored. At time = 1 h, different compounds were added to the bioreactor. DMSO was the solvent for 2-MBT and 2-MT, and EtOH for CAM. KCN is a direct inhibitor of Hmp. Solid lines represent the means of at least 3 independent experiments, and the shaded region the standard errors of the means.

Hmp is the dominant NO-detoxifying enzyme in *E. coli* under oxygenated conditions (Figure 3D) (Gardner et al., 1998; Robinson and Brynildsen, 2013; 2016b), and its expression is induced in the presence of NO (Poole et al., 1996a; Bodenmiller and Spiro, 2006). Given that 2-MBT strongly inhibited NO detoxification, compromising it to a level similar to that of Δ*hmp*, we hypothesized that 2-MBT’s cellular target was Hmp. To investigate whether 2-MBT interfered with Hmp catalysis, 2-MBT was added after NO had been cleared by exponential-phase cells, at 1 h into DPTA treatment (Figure 7). If 2-MBT were a direct inhibitor of Hmp, [NO] in the bioreactor would increase following 2-MBT addition, because the rate of enzymatic detoxification would be slowed and outcompeted by the rate of NO release by the remaining DPTA. To illustrate the effects of a direct Hmp inhibitor, 100 μM KCN was added after NO clearance. As expected, [NO] accumulated sharply after KCN treatment (Figure 7).

On the other hand, since NO detoxification requires *de novo* synthesis of Hmp upon exposure to stress, it is possible that 2-MBT could have disrupted the production of Hmp. If 2-MBT were an inhibitor of Hmp synthesis and did not impact its catalysis, [NO] would remain near zero after the addition of 2-MBT because available Hmp in the system would detoxify NO liberated from the remaining DPTA as it was released. As an example, chloramphenicol (CAM) at 100 mg/mL, which is a concentration that prevents translation (Desai and Gallivan, 2004; Adolfsen and Brynildsen, 2015), were added after cells had cleared NO, and [NO] remained close to 0 for the duration of the experiments. To ensure that ethanol (EtOH), which was used to dissolve CAM, did not contribute to experimental observations, control experiments were performed with EtOH only (Figure 7). EtOH addition did not result in any deviation from results of the DMSO control. Data from 2-MBT treatment after NO clearance show that [NO] increased, indicating that 2-MBT inhibited Hmp catalysis (Figure 7). Interestingly, when 2-MT, which inhibited NO detoxification as strongly as 2-MBT, was added after NO clearance, [NO] remained near zero (Figure 7). This suggested that 2-MT did not diminish Hmp catalysis and that the benzene ring may be important for an interaction with Hmp.

### *hmp* Promoter Activity

**FIGURE 8:**
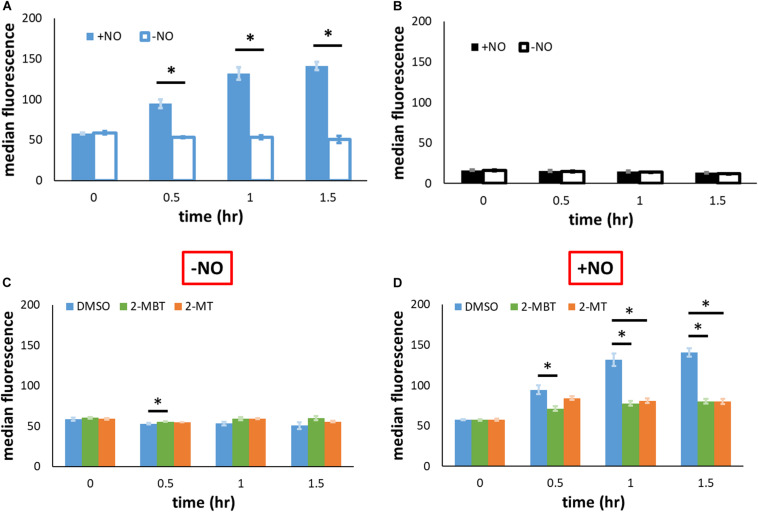
Protein output from P*_*hmp*_* in the absence and presence of NO with and without inhibitors. **(A,B)** To validate that the induction of GFP synthesis in the presence of NO was specific to the native promoter of *hmp* (P*_*hmp*_*), *gfp*_*SF*_ from the *hmp* promoter (pWC04) in *imp4213*Δ*hmp* was monitored by fluorescence in **(A)**, whereas a promoterless reporter construct (pUA66) was monitored fluorescently in **(B)**. **(C,D)** To examine the effect of compounds on the output from P*_*hmp*_*, fluorescence in *imp4213*Δ*hmp* harboring pWC04 was monitored in the absence **(C)** and presence of NO **(D)**. Values reported here are the median fluorescence of 100,000 cellular events. Solid bars represent the mean of the median from at least 3 independent experiments, and the error bars the standard errors of the means. The asterisk sign (*) indicates significant difference in fluorescence between compared groups. Statistical significance between compared pairs was determined by performing *t*-test using the *p*-value threshold of 0.05. When more than 2 conditions were compared, significance was determined by first running a one-way ANOVA test across all treatment conditions at the same time point. When the *p*-value from the ANOVA test was lower than 0.05, difference between groups were assessed by performing Tukey’s test, with a *p*-value threshold of 0.05.

As previously mentioned, another potential mechanism of inhibiting NO detoxification is by impeding Hmp synthesis. To evaluate the relevance of this mechanism, we used pWC04, which expresses *gfp*_*SF*_ from the native promoter of *hmp* (P*_*hmp*_*) on pUA66, a low-copy number plasmid (Chou and Brynildsen, 2019). We transformed this plasmid into the strain WCMV1, an *imp4213* variant that lacked *hmp* on the chromosome, to ensure consistency of NO environments between experimental conditions used (without Hmp, [NO] dynamics are indistinguishable) and fluorescence was measured to gauge protein abundance. Since P*_*hmp*_* is activated by NO, the expression of *gfp*_*SF*_ should be induced by the presence of NO. Control experiments verified that the inductive response of P*_*hmp*_* to NO was preserved in *imp4213* Δ*hmp* (Figure 8A). When a different construct, pUA66 on which *gfp* lacked a promoter, was used the fluorescence level did not increase after the introduction of NO (Figure 8B). To assess whether 2-MBT and 2-MT reduced Hmp synthesis, we monitored fluorescence accumulation following treatments with 2-MBT or 2-MT in the presence and absence of NO. As expected, when cells were not exposed to NO, there was minimal accumulation of fluorescence (Figure 8C and Supplementary Figure S6). When NO was introduced, cells treated with DMSO continuously accumulated fluorescence (Figure 8D and Supplementary Figure S6). However, in cells treated with 2-MBT and 2-MT, the induction of P*_*hmp*_* was reduced, which resulted in an ∼73% drop in the induced protein output from P*_*hmp*_*, relative to that of the DMSO control, after 1.5 h of NO treatment for both compounds. Culturability loss was ruled out as a potential contributor to the reduced protein output in WCMV1 harboring pWC04 (Supplementary Figure S7), and we also confirmed that 2-MBT and 2-MT did not impact WCMV1 growth and culturability in the absence of NO (Supplementary Figure S8). These data collectively suggest that 2-MBT and 2-MT suppress the synthesis of Hmp in response to NO.

**FIGURE 9:**
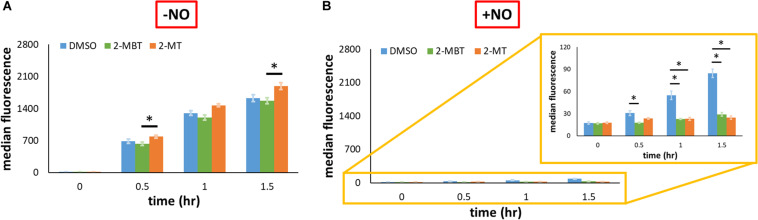
Protein output from P*_*T*5_* in the presence and absence of NO with and without inhibitors. *gfp*_*SF*_ was under the regulation of an IPTG-inducible P*_*T*5_* promoter on pUA66. At time = 0, 2 mM IPTG were added to allow the full induction of expression, and protein abundance in the absence of NO **(A)** or in its presence **(B)** was measured by fluorescence via flow cytometry. Solid bars are the mean of the median fluorescence of 100,000 cellular events from 3 biological replicates, and the error bars the standard errors of the means. Asterisk signs (*) indicate when the fluorescence between compared groups were significantly different. Statistical significance was determined by first running a one-way ANOVA test across all treatment conditions at the same time point. When the *p*-value from the ANOVA test was lower than 0.05, difference between groups were assessed by performing Tukey’s test, with a *p*-value threshold of 0.05.

To explore whether the reduction of promoter activity by 2-MBT and 2-MT was restricted to P*_*hmp*_*, we expressed *gfp*_*SF*_ from an IPTG-inducible *T*_5_ promoter on pUA66 (pWCMV1) and transformed this plasmid into WCMV1. We induced the expression of *gfp*_*SF*_ using 2 mM IPTG after treatments of 2-MBT and 2-MT and measured fluorescence in the absence and presence of NO. To validate that the effects of IPTG depended on the promoter in pWCMV1, control experiments were performed by adding 2 mM IPTG to cells containing a promoterless *gfp_mut2_* (Supplementary Figure S9), which showed that IPTG did not cause quantifiable changes to fluorescence. In the absence of NO, both 2-MBT and 2-MT did not decrease fluorescence accumulation, relative to the DMSO control (Figure 9A and Supplementary Figure S10). When NO was present, although protein synthesis was generally reduced compared to the growing control, GFP_*SF*_ abundance steadily increased in the DMSO-only sample (Figure 9B and Supplementary Figure S10), whereas both 2-MBT and 2-MT drastically stifled GFP_*SF*_ synthesis, achieving a terminal fluorescence level ∼18% and ∼10%, respectively, of that of the DMSO-only control. These results suggest that the inhibition of protein synthesis by 2-MBT and 2-MT is specific to a NO-containing environment and it is not limited to Hmp.

### Transcription From P*_*hmp*_*

**FIGURE 10:**
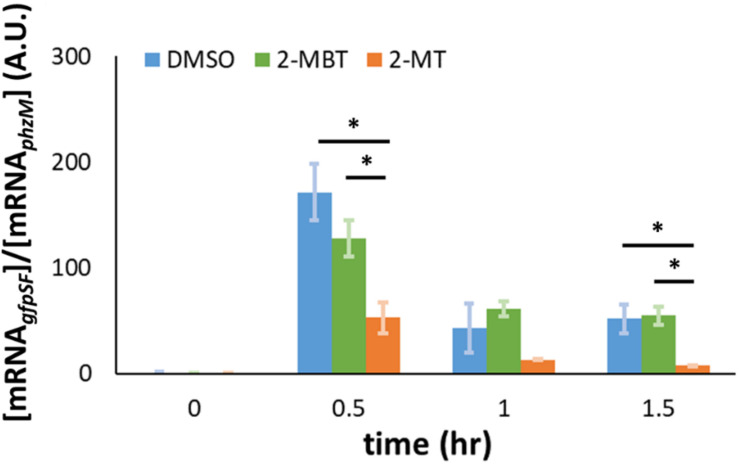
Abundance of *gfp*_*SF*_ mRNA in the presence of NO. *gfp*_*SF*_ was expressed from the native *hmp* promoter (P*_*hmp*_*) on pUA66. At time = 0, DPTA was added to achieve a final concentration of 250 μM in bioreactors containing different compounds. At time points, samples were withdrawn from the bioreactors, and the abundance of mRNA*_*gfpSF*_* in those samples was measured by qPCR. Here, [mRNA*_*gfpSF*_*] is normalized by [mRNA*_*phzM*_*] to account for losses during purification. The solid bars represent the means of at least 3 independent experiments, and the error bars the standard errors of those means. Statistical significance, indicated by the asterisk signs (*), was determined by first running a one-way ANOVA test across all treatment conditions at the same time point. When the *p*-value from the ANOVA test was lower than 0.05, difference between groups were assessed by performing Tukey’s test, with a *p*-value threshold of 0.05.

In addition to the impairment of the translational machinery, a decrease in Hmp synthesis could be caused by a reduction in transcript levels. To test whether 2-MBT and 2-MT perturb *hmp* transcript levels under NO stress, we performed qPCR to monitor the abundance of mRNA*_*gfpSF*_* expressed from P*_*hmp*_* on pWC04 in the WCMV1 background. Here, [mRNA*_*gfpSF*_*] is normalized by [mRNA*_*phzM*_*] to account for the loss of mRNA during extraction. Prior to NO treatment, transcript levels from P*_*hmp*_* remained low, which is consistent with previous studies (Chou and Brynildsen, 2019). After NO was introduced, *gfp*_*SF*_ transcript levels increased across all treatment conditions by *t* = 0.5 h (Figure 10). However, transcript levels were significantly reduced in the 2-MT treatment condition compared to both DMSO-only and 2-MBT (Figure 10). Notably, transcript levels with 2-MBT treatment did not differ significantly from the DMSO-only control. Overall, these data show that 2-MBT does not affect the expression from the *hmp* promoter appreciably, whereas 2-MT significantly reduces it.

## Discussion

NO is an important antibacterial synthesized by phagocytes (Tumer et al., 2007). Its physiological relevance is best illustrated by the increased vulnerability of mice to bacterial infections when iNOS, which is primarily responsible for NO synthesis in phagocytes, has been deleted (Hollenberg et al., 2000; Dai et al., 2003). Various pathogens have evolved strategies to deal with NO, and these defenses have been identified as crucial virulence factors. For example, using a mouse model, the deletion of *hmp* in uropathogenic *E. coli* (UPEC) resulted in a significant reduction in its ability to survive in the host (Svensson et al., 2010). The clinical relevance of *hmp* was further supported by the induction of *hmp* expression in UPEC from patients with urinary tract infections (Svensson et al., 2010). With *Yersinia pestis*, when *hmp* was removed its survivability in macrophages and virulence in rodent models were severely attenuated (Sebbane et al., 2006). The widespread importance of NO defenses for bacterial virulence makes them an appealing target for therapeutic development to fight infections. However, currently known inhibitors of these defenses have poor drug-like properties, such as human toxicity (KCN) and low permeability across bacterial outer membranes (imidazoles). These drawbacks motivated the investigation here, which seeks to identify novel inhibitors of bacterial NO defenses.

The chemical screening approach adopted in this study was inspired by previous works that used compound synergy screening (Zhang et al., 2007; Ramón-García et al., 2011; Mathews Griner et al., 2014). For example, through a synergistic screen, Zhang and colleagues successfully identified beauvericin as a synergistic partner of the fungicide ketoconazole (KTC), which increased the efficacy of KTC in a mouse model (Zhang et al., 2007). In a different screen, Ramon-Garcia and coworkers discovered numerous compounds that could synergize with spectinomycin for the treatment of mycobacteria (Ramón-García et al., 2011). Here, we first identified 45 compounds that inhibited growth in the presence of NO from 8,320 molecules, and then performed both a confirmation screen to confirm primary hit results and a counter screen in the absence of NO to distinguish compounds that were specifically deleterious in the presence of NO from those that were general growth inhibitors. A total of 17 potential NO-specific inhibitors were identified, and among them, 2-MBT was pursued further due to the magnitude and selectivity of its effects. Interestingly, 2-MBT had previously been reported to exhibit antifungal and antiviral activities (Rada et al., 1979; Kuchta et al., 1989), and the data presented here suggests that it can selectively potentiate the antibacterial effects of NO against *E. coli* (Figure 3D and Supplementary Figure S3).

By using chemical analogs of 2-MBT we explored its structure-activity relationship, and found that both sulfur atoms were crucial to the capacity of 2-MBT to inhibit NO detoxification. Moreover, even when both sulfur atoms were present, substitution of the mercapto group (−SH) with a thiomethyl (−SCH_3_) eliminated its inhibitory activity on NO detoxification (Figure 5B). The presence of the methyl group prevents disulfide bond formation. Although the thiomethyl group is also bulkier than the mercapto group, we do not think size contributes to the abolishment of inhibition because when the mercapto group was replaced by a similarly sized hydroxyl group (−OH), inhibition of NO detoxification was substantially weakened (Figure 5B). These data suggested that the ability of the mercapto group to be oxidized is important for the observed activity of 2-MBT on NO detoxification. On the contrary, the benzene ring seemed dispensable because 2-MT prohibited NO detoxification as strongly as 2-MBT. In addition, it is worth noting that thiazoles and imidazoles are chemically similar (a sulfur in place of a nitrogen); although, experiments performed with BT and 2-MBX suggest that imidazoles similar to 2-MBT would not block NO detoxification to the same extent. In support of this statement, we performed experiments with imidazole and benzimidazole under identical assay conditions and observed that 2-MT and 2-MBT were much more potent inhibitors of NO detoxification than the analogous imidazoles (Supplementary Figure S11).

Since treatment with 2-MBT or 2-MT resulted in a phenotype that resembled Δ*hmp*, we hypothesized that Hmp was the target of these compounds. To examine this hypothesis, we treated exponential-phase cells with both compounds after they had reduced the [NO] in the system to near zero. Under those conditions, DPTA continues to release NO, but [NO] stays low due to the action of Hmp. Addition of an inhibitor of Hmp catalysis would lead to an increase in [NO], which was observed for KCN, whereas if inhibition occurred through a different mechanism [NO] would stay low, such as in the case of chloramphenicol (Figure 7). The addition of 2-MBT caused a steady increase in [NO], suggesting that Hmp activity was declining (Figure 7). Interestingly, treatment with 2-MT did not change [NO] (Figure 7), which implied that 2-MT does not act through inhibition of Hmp catalysis. The difference in activity between 2-MT and 2-MBT in this assay suggested that the benzene ring of 2-MBT may be important for its interaction with Hmp. Interestingly, the hydrophobic residues of large imidazoles have previously been suggested to play a role in their interaction with and blocking of the heme pocket of Hmp (Helmick et al., 2005).

Given the differences observed for 2-MBT and 2-MT in the assay to probe inhibition of Hmp catalysis, we hypothesized that interference with Hmp synthesis, which is induced in response to NO (Poole et al., 1996b), might contribute to the abilities of 2-MT and 2-MBT to block NO detoxification. To assess this possibility, we expressed *gfp*_*SF*_ from the *hmp* promoter on a low-copy plasmid. Since P*_*hmp*_* is known to be activated by NO (Nakano et al., 2006), fluorescence did not accumulate when NO was absent (Figure 8C). In the presence of NO, the treatment of 2-MBT and 2-MT greatly impaired the inductive output from P*_*hmp*_* (Figure 8D), reducing GFP_*SF*_ accumulation by ∼3.6-fold for both compounds after 1.5 h of NO treatment, relative to the DMSO-only control. To investigate whether the inhibition of protein synthesis was limited to Hmp, we expressed *gfp*_*SF*_ from an IPTG inducible *T*_5_ promoter and used 2 mM IPTG to fully induce expression. In the absence of NO, 2-MBT and 2-MT did not repress the synthesis of GFP_*SF*_ (Figure 9A); however, when NO was present, they substantially stifled the synthesis of GFP_*SF*_, achieving an inductive fluorescence level that was approximately 5.6-fold and 9.6-fold lower for 2-MBT and 2-MT (Figure 9B), respectively, compared to that of the DMSO-only control at the terminal time point. These results suggested that 2-MBT and 2-MT not only inhibited Hmp synthesis but also arrested the production of other proteins under NO stress, whereas protein synthesis was unperturbed by these compounds in the absence of NO.

The reduction of Hmp synthesis could result from a decrease in *hmp* transcript levels. To test this, we measured the expression of *gfp*_*SF*_ from P*_*hmp*_* under an NO environment using qPCR. Our results indicated that 2-MBT does not interfere with *hmp* transcript levels, whereas 2-MT significantly reduced *hmp* transcript levels in the presence of NO compared to both 2-MBT and the DMSO-control (Figure 10). We note that the differences in transcript levels observed could have resulted from decreased transcription or enhanced mRNA degradation. These results collectively reflect the importance of the benzene ring to the biological activity of 2-MBT. Without the benzene ring, 2-MT no longer inhibited Hmp catalysis, but began to impair accumulation of transcripts expressed from the *hmp* promoter.

Thiazoles and benzothiazoles, such as 2-MT and 2-MBT, have attracted interest as therapeutic agents for a variety of applications ranging from antimicrobial and anti-inflammatory to treatment of cancer (Azam and Suresh, 2012; Martins et al., 2015; Ugwu et al., 2018; Tariq et al., 2019). Clinical successes with thiazoles include dabrafenib for treating advanced melanoma and metastatic lung cancer (Ballantyne and Garnock-Jones, 2013; Odogwu et al., 2018), riluzole for delaying the progression of amyotrophic lateral sclerosis (ALS) (Miller et al., 2002), and pramipexole for treating Parkinson’s disease (Constantinescu, 2008). In addition, there are numerous thiazoles and benzothiazoles undergoing clinical trials for the treatment of Alzheimer’s disease (Okamoto et al., 2018), depression (ClinicalTrials.gov, 2019c), asthma (ClinicalTrials.gov, 2019a), and melanoma (Cerezo et al., 2016; ClinicalTrials.gov, 2019b, d). Collectively, these efforts show that the thiazole and benzothiazole scaffold is amenable to therapeutic applications. The findings reported here suggest that those scaffolds hold potential for the potentiation of NO against bacteria. However, important questions remain regarding the properties of 2-MBT and 2-MT, such as their ability to cross bacterial membranes and their proclivity to be expelled by bacterial multi-drug efflux pumps. Indeed, additional structure-activity experiments need to be performed, especially in light of the fact that both 2-MBT and 2-MT are irritants (Hudson and Dotson, 2014; National Center for Biotechnology Information, 2020), and the mechanisms of action need to be fleshed out at the molecular level. Despite these future milestones, identification of compounds that impair bacterial propagation and defenses selectively in the presence of NO lends confidence to the potential of bacterial NO defenses as anti-infective targets.

## Data Availability Statement

This article contains previously unpublished data. The name of the repository and accession number(s) are not available.

## Author Contributions

WC, MV, HK, and MB conceived and planned the experiments and contributed to the interpretation of the results. WC and MV carried out the experiments. MV and HK contributed to the sample preparation for the screen. WC and MB took the lead in writing the manuscript. All the authors provided critical feedback and helped shape the research, analysis and manuscript.

## Conflict of Interest

The authors declare that the research was conducted in the absence of any commercial or financial relationships that could be construed as a potential conflict of interest.
