# Supplementary material for: Synergy Screening Identifies a Compound That Selectively Enhances the Antibacterial Activity of Nitric Oxide
Source: Front Bioeng Biotechnol. 2020 Aug 25;8:1001. doi: 10.3389/fbioe.2020.01001 (PMC7477088; doi:10.3389/fbioe.2020.01001)
Supplement: Supplementary file 1 [file Image_1.PDF]

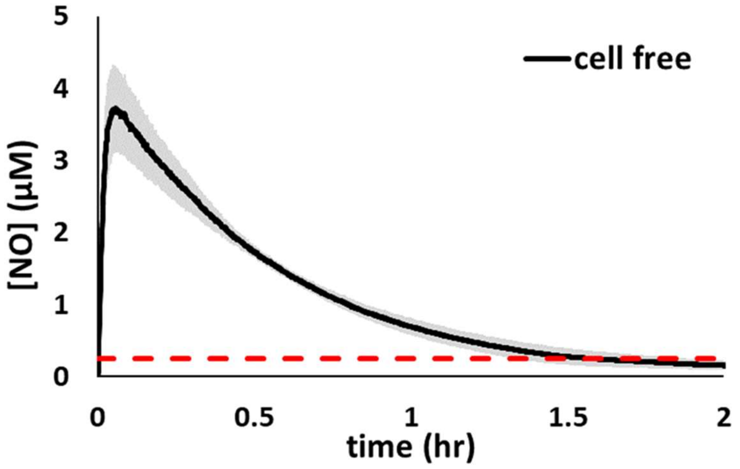

Fig. S1 Cell-free [NO] profile when PAPA was treated.

34 μM of PAPA were treated at t = 0 to a bioreactor in the absence of cells. [NO] in the solution was measured continuously using an NO probe. The bold solid line represents the average [NO] measurements from 3 independent replicates, and the light shadow around the bold line the error of those measurements. The red dashed line represents the threshold of [NO] clearance at 0.2 μM, below which NO is considered depleted in the bioreactor.
