# Supplementary material for: Synergy Screening Identifies a Compound That Selectively Enhances the Antibacterial Activity of Nitric Oxide
Source: Front Bioeng Biotechnol. 2020 Aug 25;8:1001. doi: 10.3389/fbioe.2020.01001 (PMC7477088; doi:10.3389/fbioe.2020.01001)
Supplement: Supplementary file 2 [file Image_2.PDF]

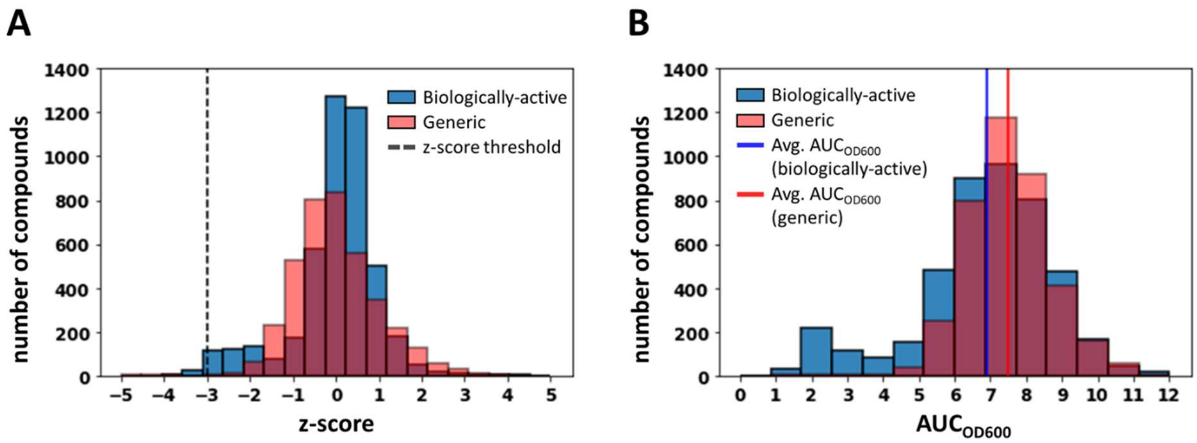

Fig. S2 Distribution of z-scores and AUC$_{OD600}$ by the sub-libraries.

Histograms of z-scores and AUC$_{OD600}$ from the initial screen by the sub-libraries are plotted. Vertical grey line in (A) represents the threshold of -3 used in this study for hit identification, and the solid vertical blue and red lines in (B) represent the average AUC$_{OD600}$ among compounds from the biologically-active library and the generic library, respectively.
