# Supplementary material for: Synergy Screening Identifies a Compound That Selectively Enhances the Antibacterial Activity of Nitric Oxide
Source: Front Bioeng Biotechnol. 2020 Aug 25;8:1001. doi: 10.3389/fbioe.2020.01001 (PMC7477088; doi:10.3389/fbioe.2020.01001)
Supplement: Supplementary file 3 [file Image_3.PDF]

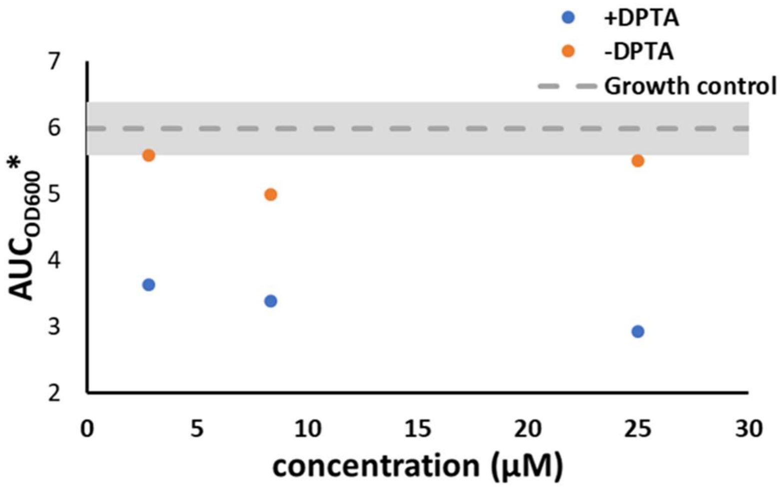

Fig. S3 AUC_{OD600} of 2-MBT across various concentrations.

Growth in various concentrations of 2-MBT in the presence or absence of NO was quantified as AUC_{OD600}. The initial OD_{600} used to inoculate the bioreactor was 0.05. In this case, non-growing cultures would still have a positive AUC_{OD600} contributed by integrating 0.05 over time. To account for this, the AUC_{OD600}* reported here was calculated by subtracting the baseline AUC_{OD600} from non-growing cultures. The grey dashed line represents the average AUC_{OD600} measured in WT grown in the absence of NO and 2-MBT, and the lighter shade around the grey line represents the standard error of the mean.
