# Supplementary material for: Synergy Screening Identifies a Compound That Selectively Enhances the Antibacterial Activity of Nitric Oxide
Source: Front Bioeng Biotechnol. 2020 Aug 25;8:1001. doi: 10.3389/fbioe.2020.01001 (PMC7477088; doi:10.3389/fbioe.2020.01001)
Supplement: Supplementary file 4 [file Image_4.PDF]

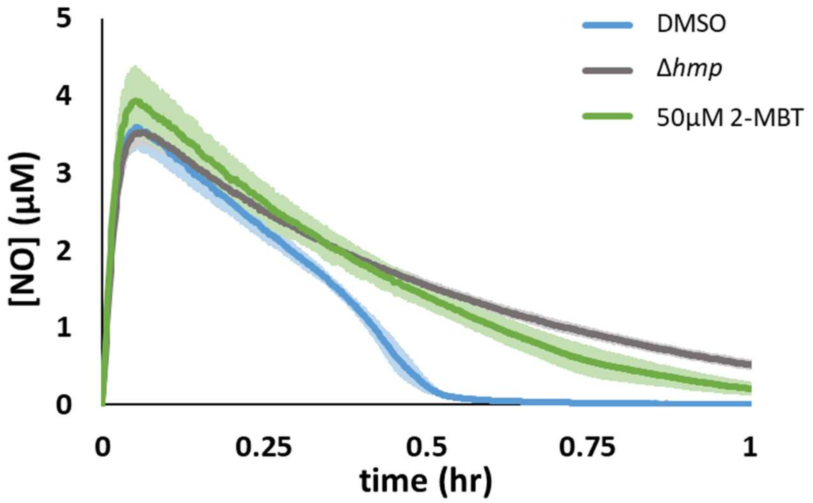

Fig. S4 Assessment of the inhibitory effects of 2-MBT using a different NO donor.

[NO] dynamics in cell cultures in the presence of 2-MBT or DMSO using a different NO donor (PAPA). Dynamics were different from those in Fig. 3D due to different release kinetics of DPTA and PAPA. Hmp deletion mutant represents [NO] dynamics in the absence of cellular NO consumption. The bold solid lines represent the averages from 3 independent replicates, and the light shades around the lines represent the standard errors of the means.
