# Supplementary material for: Synergy Screening Identifies a Compound That Selectively Enhances the Antibacterial Activity of Nitric Oxide
Source: Front Bioeng Biotechnol. 2020 Aug 25;8:1001. doi: 10.3389/fbioe.2020.01001 (PMC7477088; doi:10.3389/fbioe.2020.01001)
Supplement: Supplementary file 5 [file Image_5.PDF]

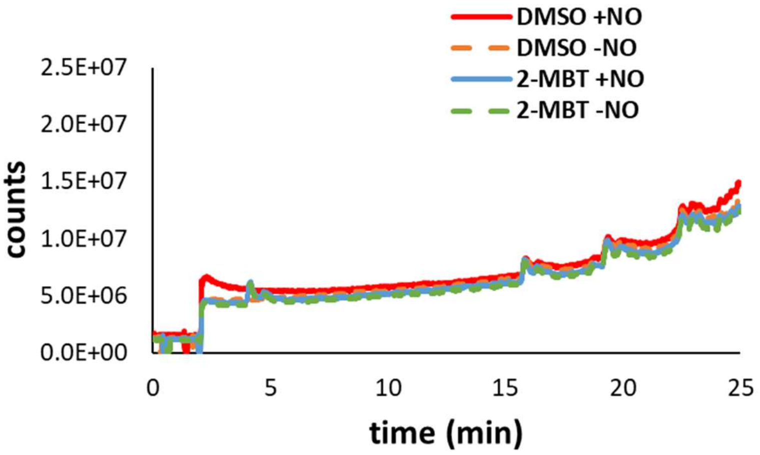

Fig. S5 Representative chromatograms from LC-MS analysis of samples from cell-free experiments.

34 µM of PAPA or 34.7 µL of 10 mM NaOH were added to a cell-free bioreactor containing MOPS and 50 µM of 2-MBT or DMSO. After the depletion of NO (~ 2 hr after PAPA treatment), composition of the bioreactor was analyzed using LC-MS. Representative total-ion current chromatogram (TIC) of 3 replicate experiments are plotted here.
