# Supplementary material for: Synergy Screening Identifies a Compound That Selectively Enhances the Antibacterial Activity of Nitric Oxide
Source: Front Bioeng Biotechnol. 2020 Aug 25;8:1001. doi: 10.3389/fbioe.2020.01001 (PMC7477088; doi:10.3389/fbioe.2020.01001)
Supplement: Supplementary file 6 [file Image_6.PDF]

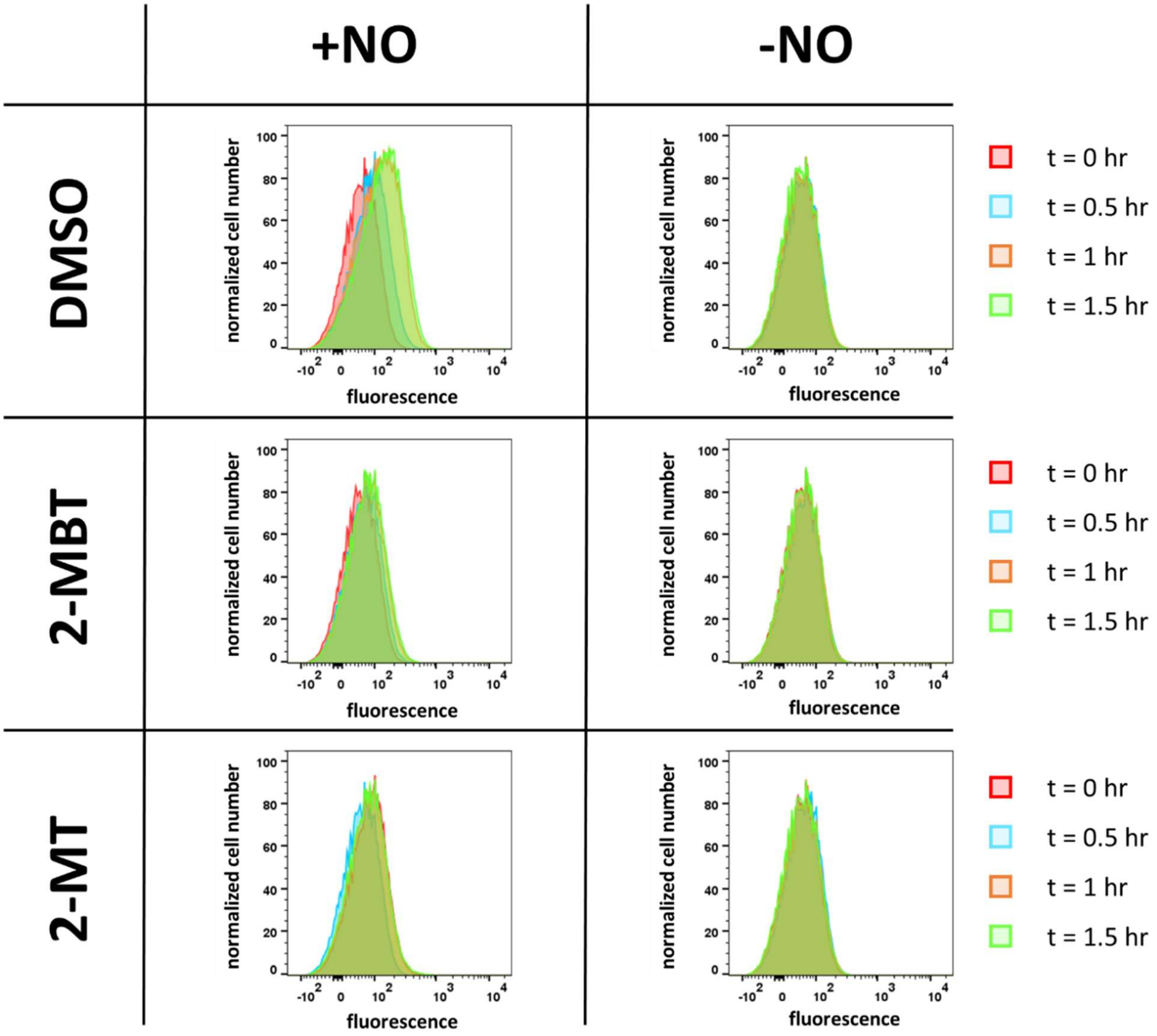

Fig. S6 Representative histograms of fluorescence from *imp4213 Δhmp* containing pWC04.

Fluorescence from *imp4213 Δhmp* harboring pWC04 under different treatment conditions was measured using flow cytometry. Presented here are representative samples from 1 of 3 independent, biological replicates.
