# Supplementary material for: Synergy Screening Identifies a Compound That Selectively Enhances the Antibacterial Activity of Nitric Oxide
Source: Front Bioeng Biotechnol. 2020 Aug 25;8:1001. doi: 10.3389/fbioe.2020.01001 (PMC7477088; doi:10.3389/fbioe.2020.01001)
Supplement: Supplementary file 7 [file Image_7.PDF]

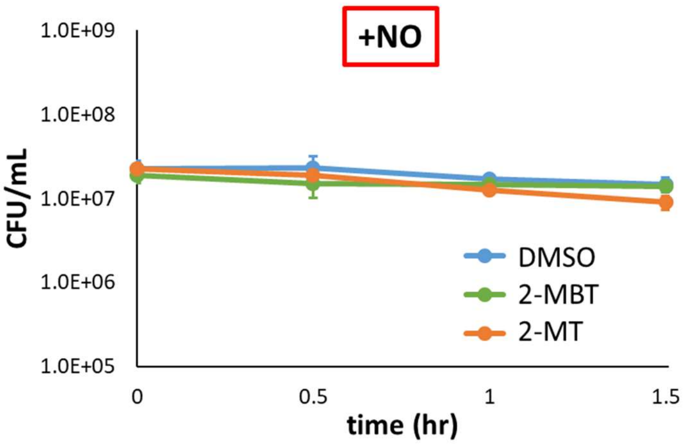


**Fig. S7 Culturability of *imp4213 Δhmp* harboring pWC04 during NO stress.**

Culturability of *imp4213 Δhmp* containing pWC04 in the presence of NO was measured by counting CFU/mL over time. The solid dots are the average CFU/mL from 3 independent replicates, and the error bars are the standard errors of the means.
