# Supplementary material for: Synergy Screening Identifies a Compound That Selectively Enhances the Antibacterial Activity of Nitric Oxide
Source: Front Bioeng Biotechnol. 2020 Aug 25;8:1001. doi: 10.3389/fbioe.2020.01001 (PMC7477088; doi:10.3389/fbioe.2020.01001)
Supplement: Supplementary file 8 [file Image_8.PDF]

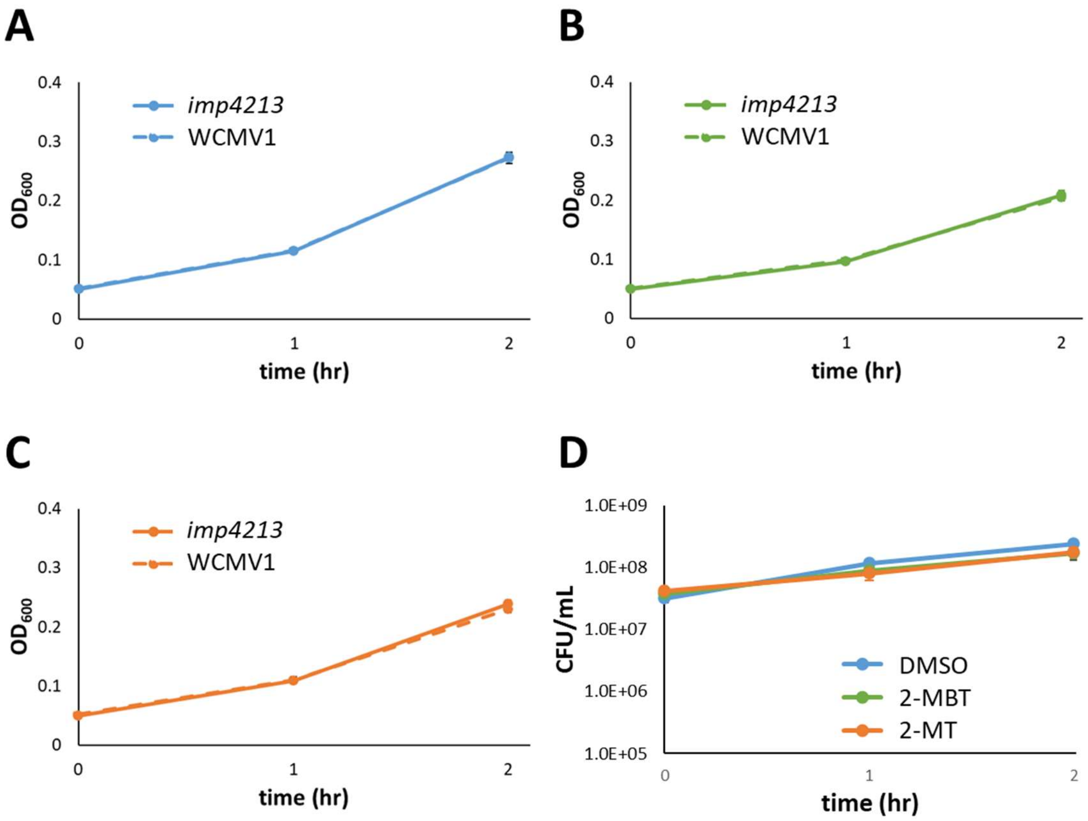

Fig. S8 Effects of 2-MBT and 2-MT on *imp4213* Δ*hmp* in the absence of NO.

Growth of *imp4213* Δ*hmp* (WCMV1) in the presence of DMSO (A), 50 μM of 2-MBT (B), or 50 μM of 2-MT (C) was monitored by measuring OD_{600} every hour and compared to that of *imp4213* under identical conditions. Culturability of *imp4213* Δ*hmp* was also quantified by measuring CFU in the presence of different compounds (D). The solid dots are the averages of at least 3 biological replicates, and the error bars the standard errors of the associated measurements.
