# Supplementary material for: Synergy Screening Identifies a Compound That Selectively Enhances the Antibacterial Activity of Nitric Oxide
Source: Front Bioeng Biotechnol. 2020 Aug 25;8:1001. doi: 10.3389/fbioe.2020.01001 (PMC7477088; doi:10.3389/fbioe.2020.01001)
Supplement: Supplementary file 9 [file Image_9.PDF]

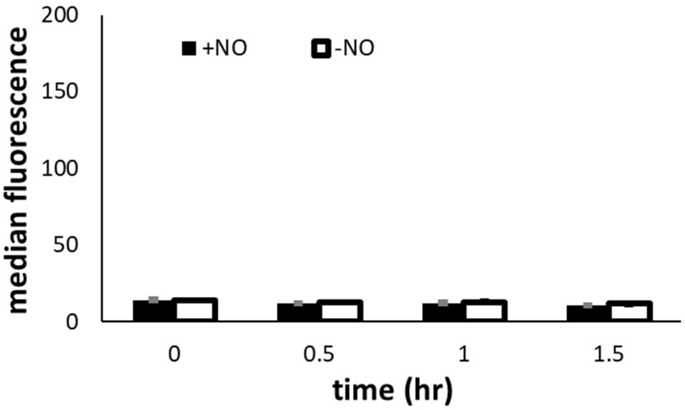


**Fig. S9 Effect of IPTG on fluorescence measurements.**

To ensure that IPTG did not affect fluorescence measurements in the absence of the IPTG-inducible *T5* promoter (Fig. 10), a promoterless reporter construct (pUA66) was transformed into *imp4213* Δ*hmp*. At t = 0, 2 mM IPTG was added, and fluorescence was measured in the presence and absence of NO. The bars are the means of the median fluorescence measurement among 100,000 cellular events from 3 biological replicates, whereas the error bars reflect the errors of the means.
