# Supplementary material for: Synergy Screening Identifies a Compound That Selectively Enhances the Antibacterial Activity of Nitric Oxide
Source: Front Bioeng Biotechnol. 2020 Aug 25;8:1001. doi: 10.3389/fbioe.2020.01001 (PMC7477088; doi:10.3389/fbioe.2020.01001)
Supplement: Supplementary file 10 [file Image_10.PDF]

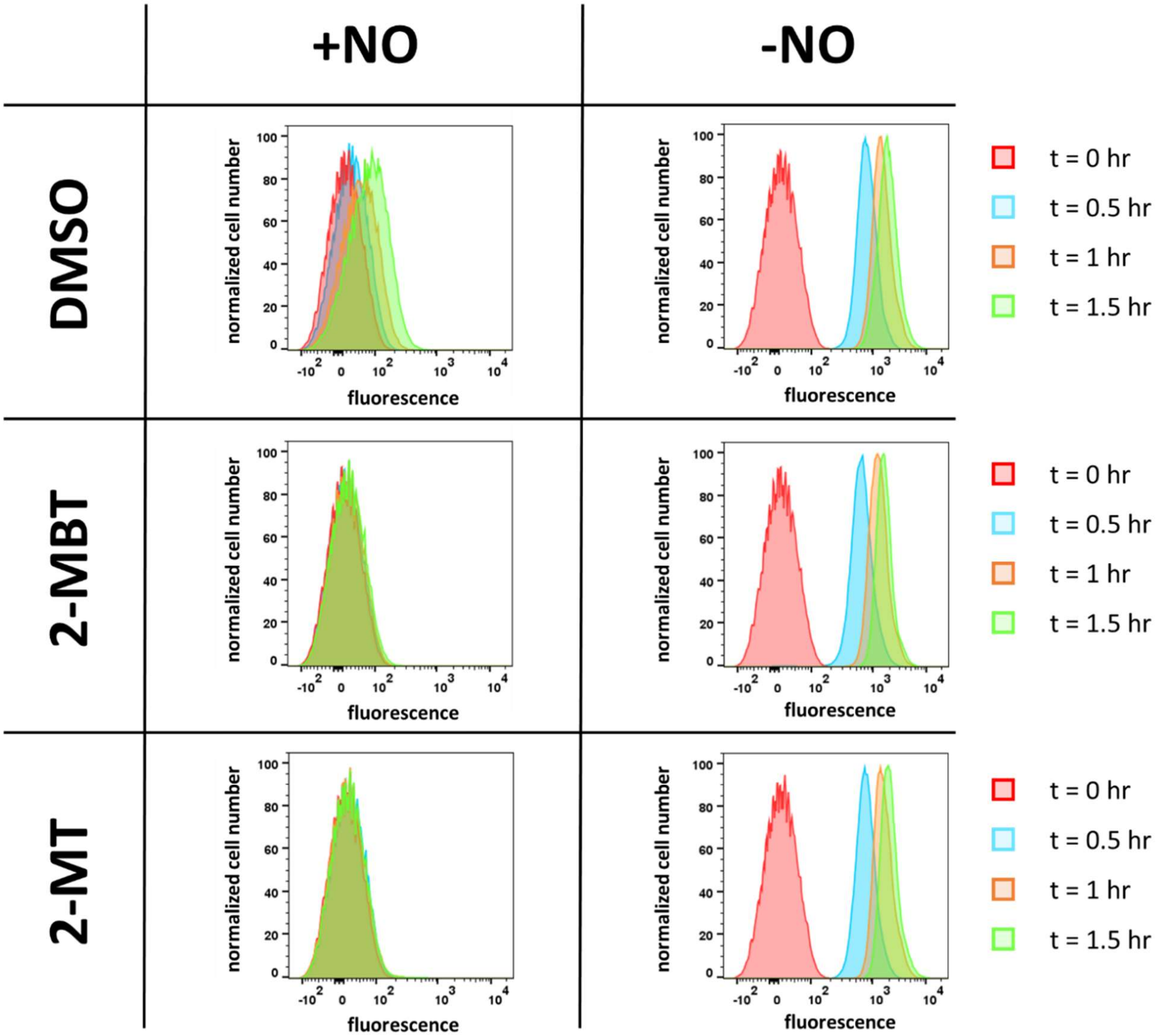

Fig. S10 Representative histograms of fluorescence from of ***imp4213 Δhmp*** **containing pWCMV1.**

Fluorescence from of *imp4213 Δhmp* harboring pWCMV1 under various treatment conditions was measured using flow cytometry. To induce the expression of gfp_{SF} from P_{T5}, 2 mM IPTG was added at t = 0. Presented here are representative samples from 1 of 3 independent, biological replicates.
