# Supplementary material for: Synergy Screening Identifies a Compound That Selectively Enhances the Antibacterial Activity of Nitric Oxide
Source: Front Bioeng Biotechnol. 2020 Aug 25;8:1001. doi: 10.3389/fbioe.2020.01001 (PMC7477088; doi:10.3389/fbioe.2020.01001)
Supplement: Supplementary file 11 [file Image_11.PDF]

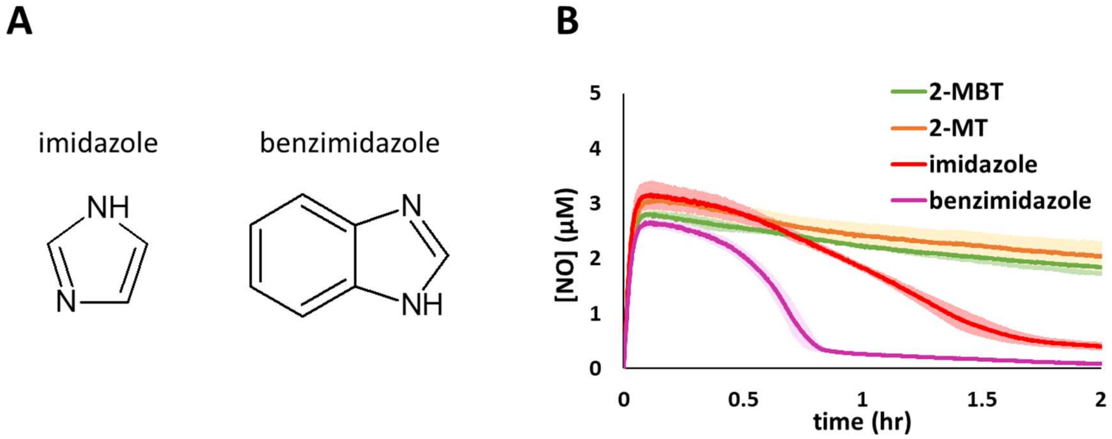

Fig. S11 Inhibitory effects of imidazoles on NO detoxification in *imp4213*.

A) Structures of imidazole and benzimidazole were drawn using ACD/ChemSketch (1). B) Before DPTA treatment, 50 µM of imidazole or benzimidazole were treated to exponential-phase *imp4213* in the bioreactor. [NO] in the bioreactor was measured continuously, and the [NO] profiles were compared to those resulting from the treatment of the same concentration of 2-MBT and 2-MT. The bold lines are the averages of at least 3 biological replicates, and the lighter shades around the lines the standard errors of the means.

References

1. I. Advanced Chemistry Development: ACD/ChemSketch. In, Toronto, ON, Canada (2019)
