# Supplementary material for: Synergy Screening Identifies a Compound That Selectively Enhances the Antibacterial Activity of Nitric Oxide
Source: Front Bioeng Biotechnol. 2020 Aug 25;8:1001. doi: 10.3389/fbioe.2020.01001 (PMC7477088; doi:10.3389/fbioe.2020.01001)
Supplement: Supplementary file 12 [file Table_1.DOCX]

**Table S1. Strain and plasmid table.**

| **Strain** | **Genotype** | **Reference** |
| --- | --- | --- |
| *imp4213* | *E. coli* MG1655, *lptD*4213 | (1) |
| WCMV1 | *imp4213*, Δ*hmp*::*camR* | This study |
| **Plasmid** | **Genotype** | **Source or Reference** |
| pUA66 | Vector, SC101ori, *kanR*, *gfpmut2* | (2) |
| pWCMV1 | pUA66 P*_T5_*-*gfp_SF_* | This study |
| pWC04 | pUA66 P*_hmp_*-*gfp_SF_* | (3) |

**References**

1. N. Ruiz, B. Falcone, D. Kahne and T. J. Silhavy: Chemical conditionality: a genetic strategy to probe organelle assembly. *Cell*, 121(2), 307-17 (2005) doi:10.1016/j.cell.2005.02.014

2. A. Zaslaver, A. Bren, M. Ronen, S. Itzkovitz, I. Kikoin, S. Shavit, W. Liebermeister, M. G. Surette and U. Alon: A comprehensive library of fluorescent transcriptional reporters for Escherichia coli. *Nat Methods*, 3(8), 623-8 (2006) doi:10.1038/nmeth895

3. W. K. Chou and M. P. Brynildsen: Loss of DksA leads to multi-faceted impairment of nitric oxide detoxification by Escherichia coli. *Free Radic Biol Med*, 130, 288-296 (2019) doi:10.1016/j.freeradbiomed.2018.10.435
