# Supplementary material for: Synergy Screening Identifies a Compound That Selectively Enhances the Antibacterial Activity of Nitric Oxide
Source: Front Bioeng Biotechnol. 2020 Aug 25;8:1001. doi: 10.3389/fbioe.2020.01001 (PMC7477088; doi:10.3389/fbioe.2020.01001)
Supplement: Supplementary file 13 [file Table_2.DOCX]

**Table S2. Primer table.**

| Primer | Sequence (5’ to 3’) | Description | Reference |
| --- | --- | --- | --- |
| 1 | CCGAATCATTGTGCGATAACA | External forward of *hmp*, used with primers 3 and 4 to detect the presence or deletion of *hmp*. | (1) |
| 2 | TCCCTTTACTGGTGGAAACG | Internal forward of *hmp*, used with primer 3 to detect the presence or deletion of *hmp*. | (1) |
| 3 | CACGCCCAGATCCACTAACT | Internal reverse of *hmp*, used with primers 1 and 2 to detect the presence or deletion of *hmp*. | This study |
| 4 | AAGCATCTGCCGACATGGAA | Internal reverse of *camR*, used with primer 1 to detect the presence or deletion of *hmp*. | This study |
| 5 | GACGGGAACTACAAGACACG | Internal primer of *gfp_SF_*, used with primer 6 during PCR to quantify *gfp_SF_* expression. | This study |
| 6 | TTGTTTGTCTGCCATGATGTA | Internal primer of *gfp_SF_*, used with primer 5 during PCR to quantify *gfp_SF_* expression. | This study |
| 7 | CGGCGAAGACTTCTACAGCTA | Internal primer of *phzM*, used with primer 8 during PCR to quantify *phzM* mRNA. | (1) |
| 8 | CAGGATGGCCTTGGTCAAT | Internal primer of *phzM*, used with primer 7 during PCR to quantify *phzM* mRNA. | (1) |

**References**

1. W. K. Chou and M. P. Brynildsen: Loss of DksA leads to multi-faceted impairment of nitric oxide detoxification by Escherichia coli. *Free Radic Biol Med*, 130, 288-296 (2019) doi:10.1016/j.freeradbiomed.2018.10.435
